# Supplementary figures and images for: The hepatitis E virus capsid protein ORF2 counteracts cell-intrinsic antiviral responses to enable persistent replication in cell culture
Source: PLoS Pathog. 2025 Sep 22;21(9):e1013516. doi: 10.1371/journal.ppat.1013516 (PMC12478880; doi:10.1371/journal.ppat.1013516)

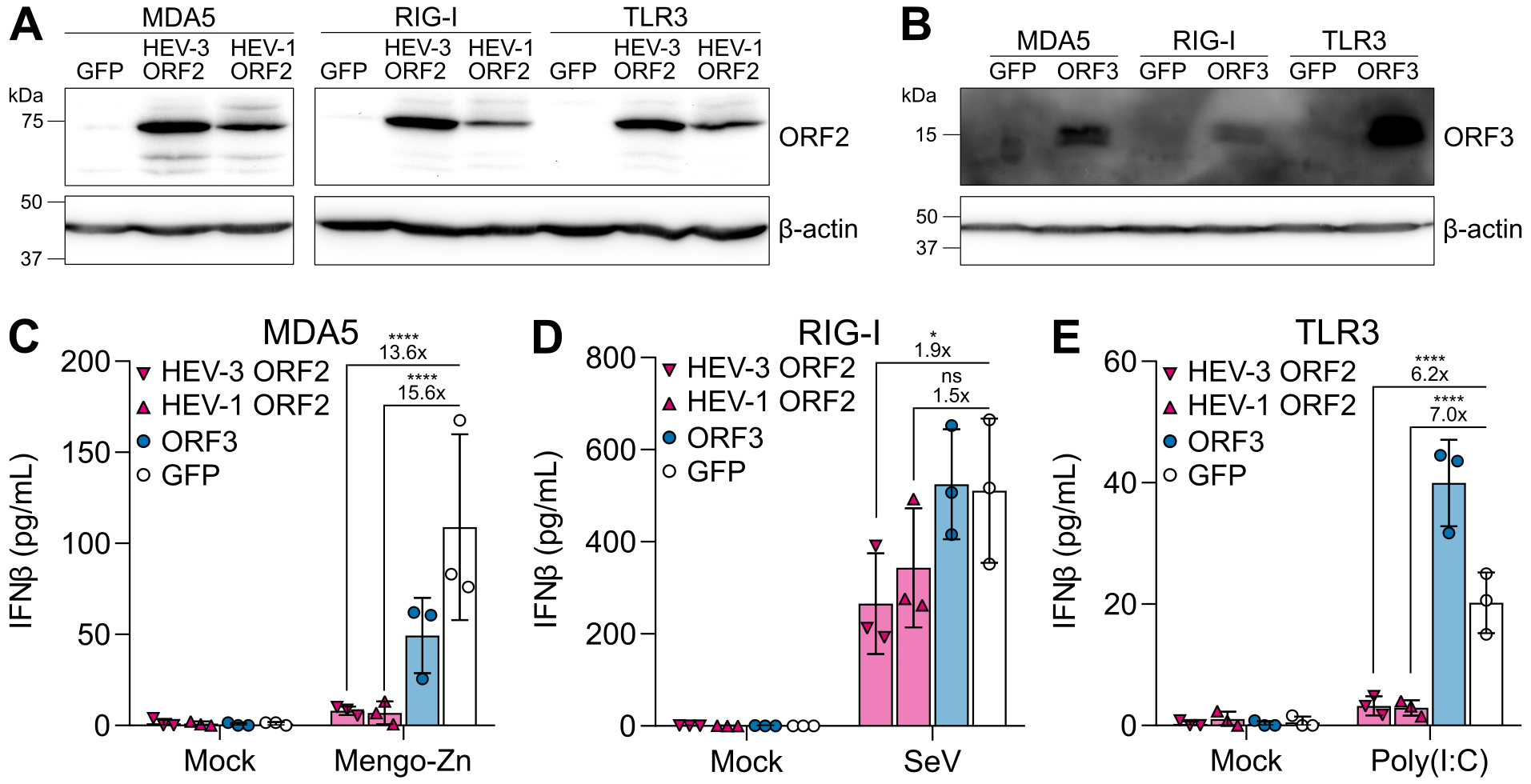

Supplement: S1 Fig — (A) A549 cells harboring knockouts of the PRRs RIG-I and MDA5 and ectopically expressing a single PRR (MDA5, RIG-I, or TLR3) together with either HEV-3 ORF2, HEV-1 ORF2, ORF3, or GFP were analyzed for ORF2 or (B) ORF3 protein expression by Western blot, together with the loading control β-actin. (C) A549-derived cell lines were challenged with either Mengo-Zn virus at MOI 1, (D) Sendai virus (SeV) at MOI 0.75, or (E) 50 µg/mL poly(I:C) supernatant feeding for 24 h. Supernatant was collected and IFNβ was measured by enzyme-linked immunosorbent assay (ELISA). Numbers indicate fold reductions compared to GFP. Data shown mean ± SD of n = 3 independent biological experiments. Statistical analysis was performed using two-way ANOVA. *, p < 0.05; ****: p < 0.0001; ns, non-significant. (TIFF) [file ppat.1013516.s001.tiff]

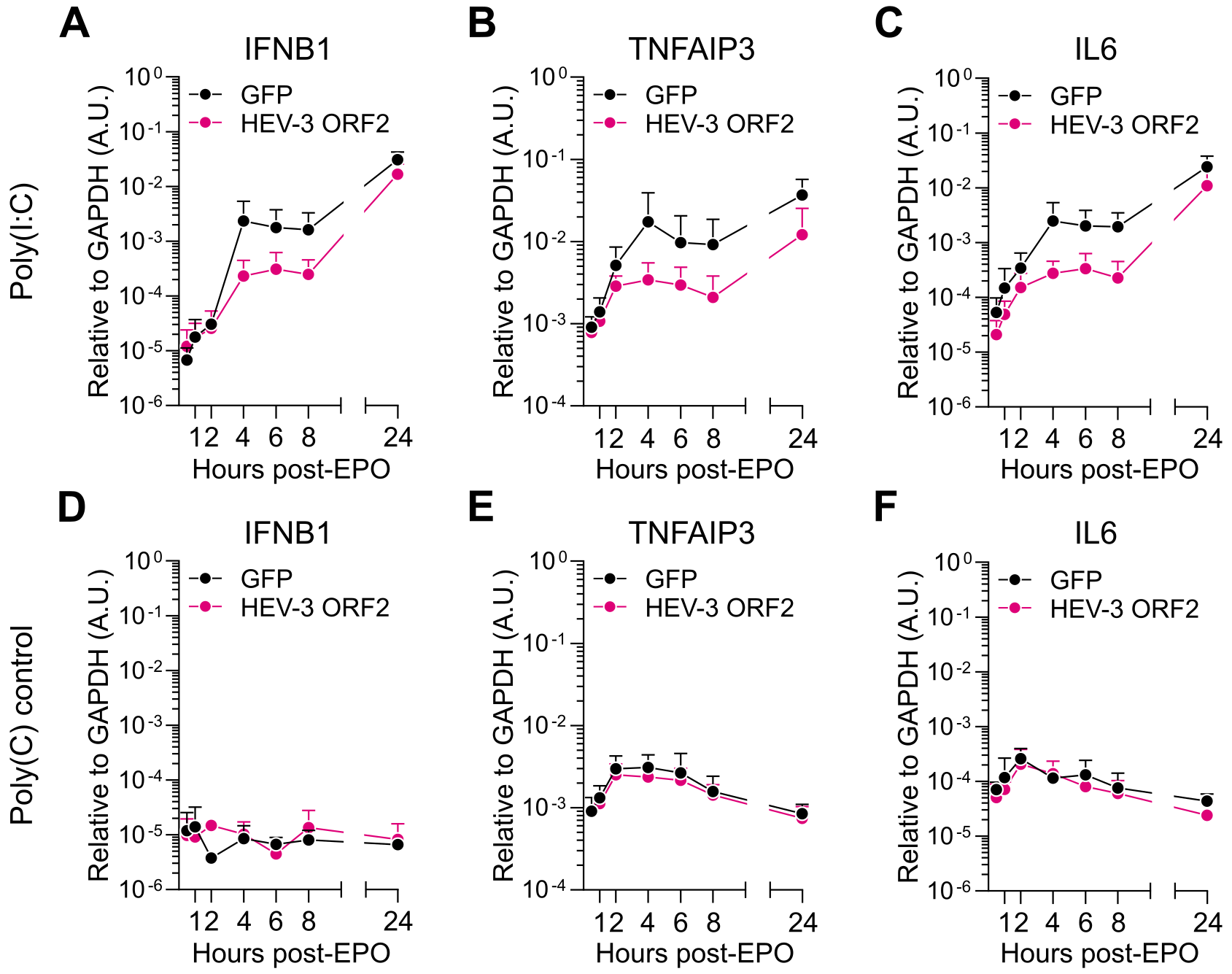

Supplement: S2 Fig — (A) A549 cells harboring knockouts of the PRRs RIG-I and MDA5 and ectopically expressing MDA5 and either GFP or HEV-3 ORF2 were electroporated with poly(I:C) and analyzed for IFNB1, (B) TNFAIP3, and (C) IL6 expression at indicated time points, relative to the housekeeping gene GAPDH using the 2-ΔCt method. (D) As a control, A549-derived cells were electroporated with poly(C) and analyzed for IFNB1, (E) TNFAIP3, and (F) IL6 expression at indicated time points, relative to the housekeeping gene GAPDH using the 2-ΔCt method. Data shown mean ± SD of n = 3 independent biological experiments. (TIFF) [file ppat.1013516.s002.tiff]

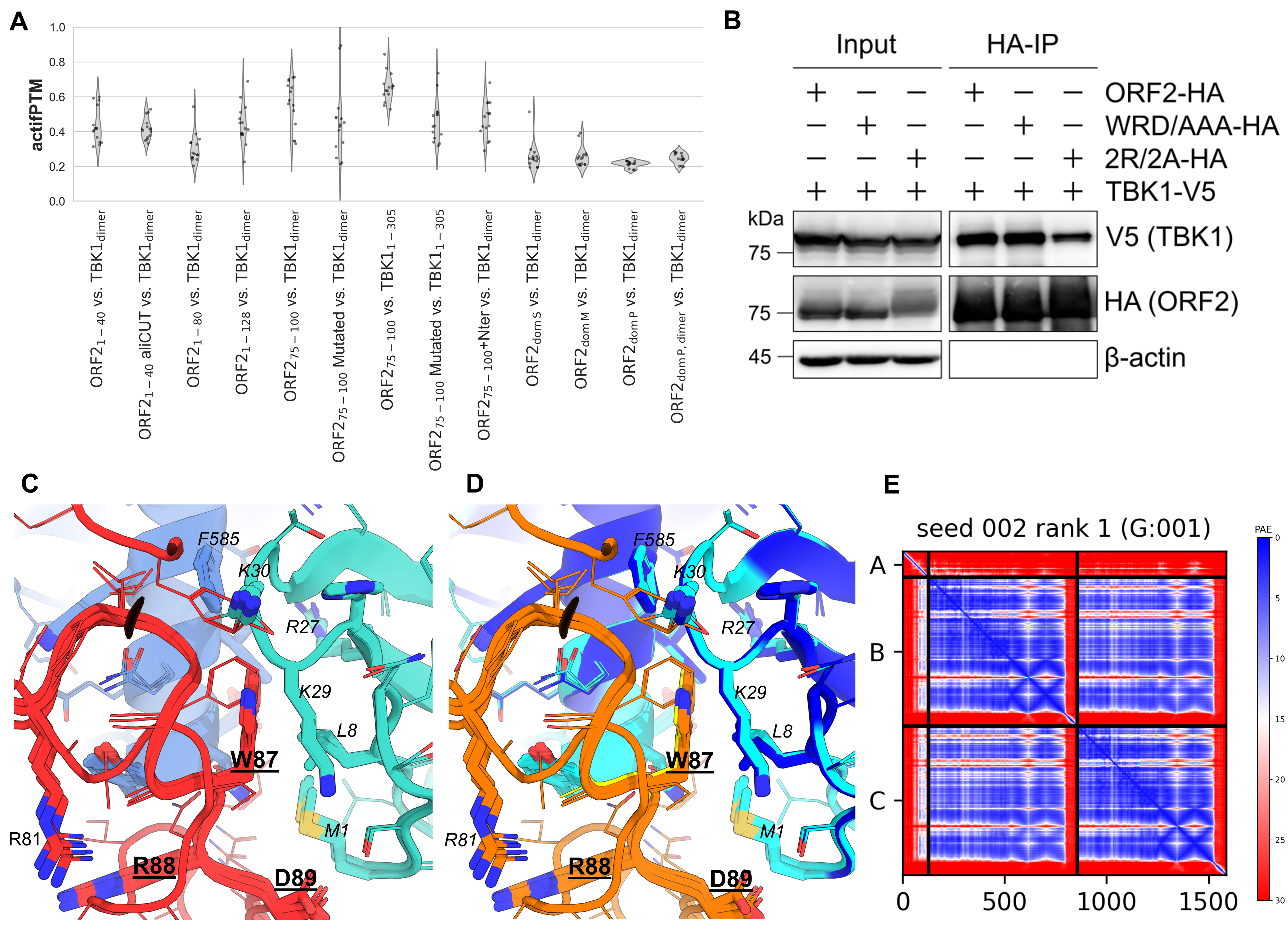

Supplement: S3 Fig — (A) actifPTM scores assessing predicted interactions between segments of ORF2 and TBK1. Higher scores indicate stronger predicted interactions. (B) HEK293T cells were transfected with ORF2-HA, ORF2-WRD/AAA-HA, or ORF2-2R/2A-HA and V5-tagged TBK1 and lysed 24 h post-transfection. Anti-HA co-IP and WB analysis for TBK1 (anti-V5 staining), ORF2 (anti-HA staining), and β-actin were performed. Representative blot of n = 2 independent biological experiments. (C–D) Structural analysis of the top five ranked models for the interaction between ORF2 residues 1–128 (red) and a TBK1 dimer. Panel C highlights the molecular environment surrounding ORF2 W87, color-coded by domain (TBK1 kinase domain in turquoise, C-terminal domain in blue). Panel D depicts the same region colored according to pLDDT scores, reflecting model confidence (orange – very low; yellow – low; cyan – high; blue – very high). Modeling convergence of the models and the chemical environment of W87 and R88 suggest potential for stabilizing cation-π interactions. (E) Predicted Aligned Error (PAE) for the highest-ranking ORF2 1–128 vs. TBK1 dimer model. Low PAE values in the N-terminal region of ORF2 1–128, particularly with respect to TBK1 chains B and C, indicate a likely accurate placement of this region relative to the TBK1 monomers. (TIF) [file ppat.1013516.s003.tif]

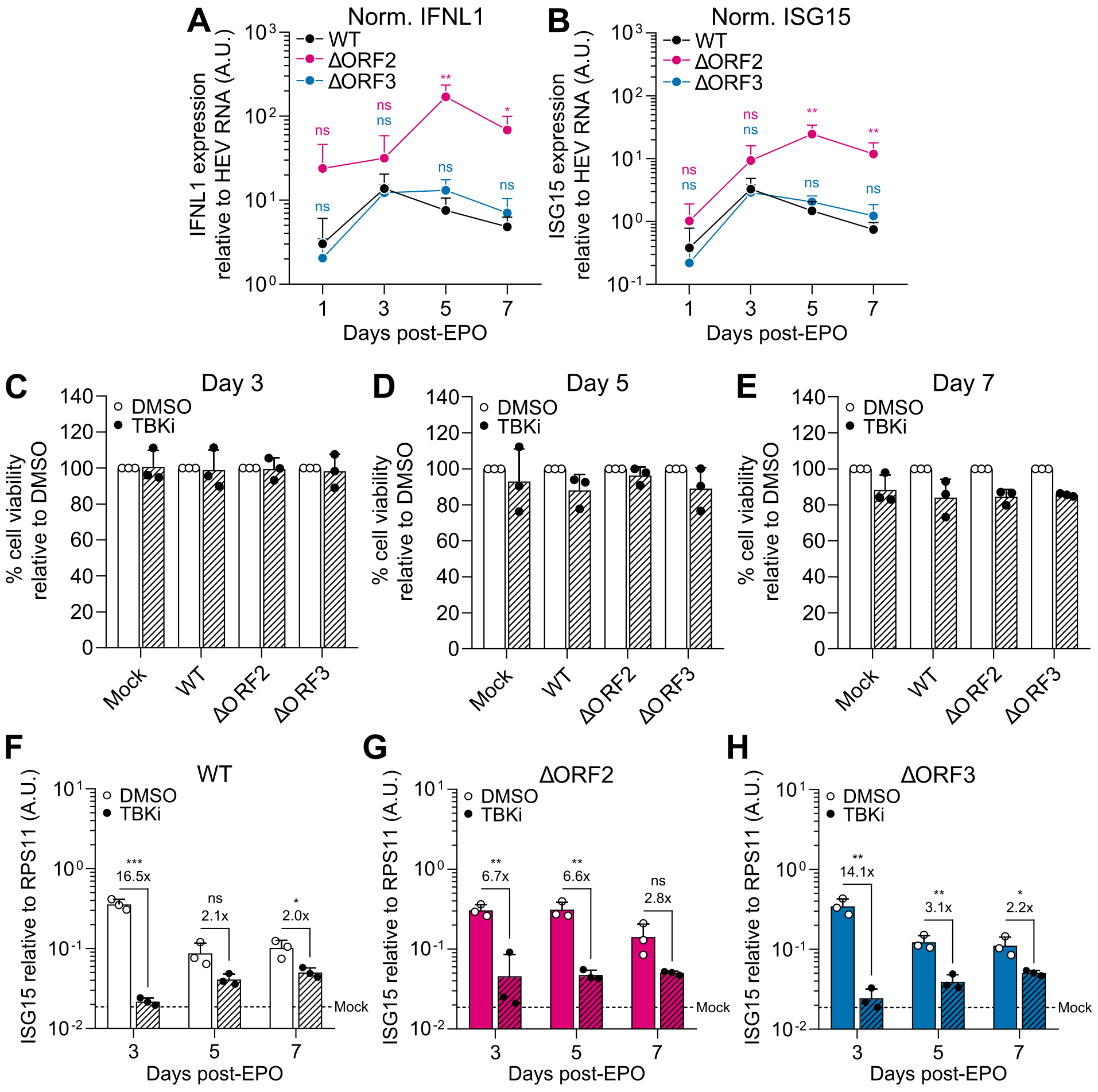

Supplement: S4 Fig — (A–B) Normalized data of Fig 2C and 2D over HEV RNA: Expression of (A) IFNL1 and (B) ISG15 upon EPO of HepG2/C3A cells with HEV WT, ΔORF2, or ΔORF3 were determined relative to the housekeeping gene RPS11 and normalized to HEV RNA using the 2-ΔΔCt method. Data show mean ± SD of n = 3 independent biological experiments. Statistical analysis was performed using one-way ANOVA of each time point independently, and comparisons to WT are indicated above the respective time points in the corresponding colors. *: p < 0.05; **: p < 0.01; ns, non-significant. A.U., arbitrary units; norm., normalized. (C) HepG2/C3A cells were either mock-electroporated or electroporated with HEV WT, ΔORF2, or ΔORF3 RNA and additionally treated with 6 µM of the TBK1 inhibitor BX795 (TBKi) or respective DMSO vehicle control 48 h prior to the time point of harvest. Cell viability was determined on days 3, (D) 5, and (E) 7 post-EPO using the CellTiter 96 AQueous One Solution Cell Proliferation Assay (Promega). Data was normalized to the respective DMSO controls. Data show mean ± SD of n = 3 independent biological experiments. (F) Cell lysates from Fig 2E–G were analyzed for ISG15 expression relative to RPS11 using the 2-ΔCt method. The dashed line indicates the mean of ISG15 basal expression in mock-electroporated cells under TBKi treatment across days 3, 5, and 7. Data show mean ± SD of n = 3 independent biological experiments. *: p < 0.05; **: p < 0.01; ***: p < 0.001; ns, non-significant. (TIFF) [file ppat.1013516.s004.tiff]

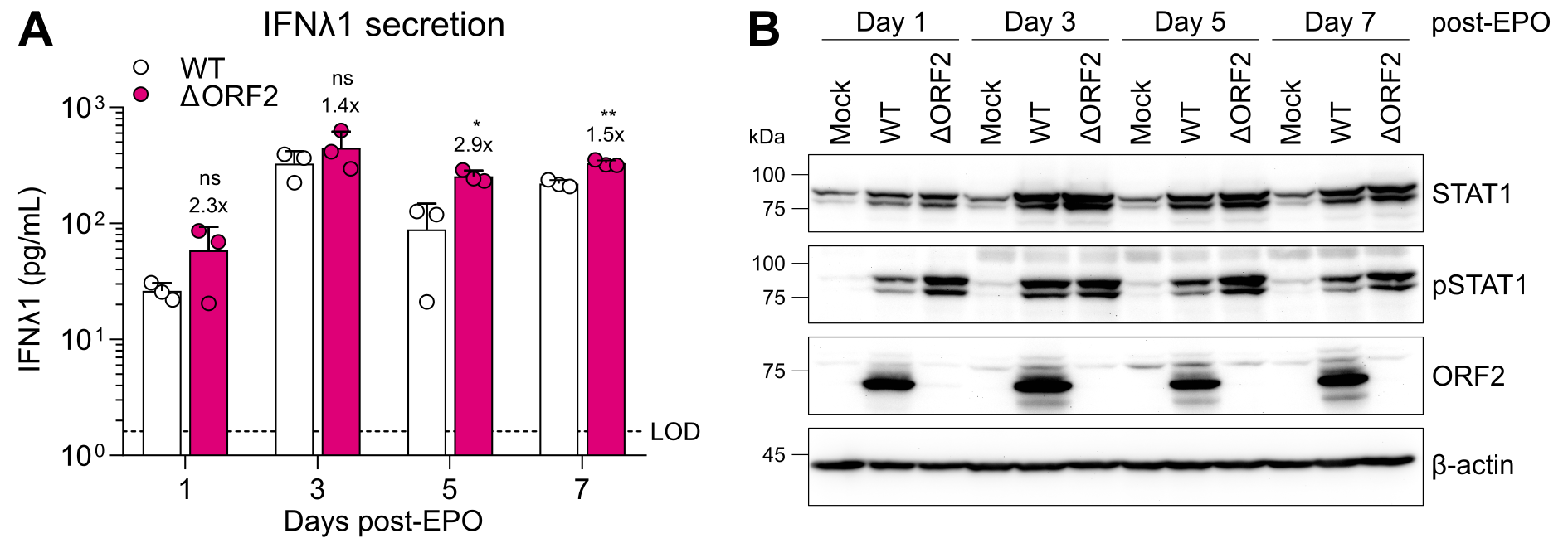

Supplement: S5 Fig — (A) HepG2/C3A cells were electroporated with HEV WT or ΔORF2 and mixed 1:1 with mock-electroporated cells. Supernatants were analyzed for secreted IFNλ1 protein on indicated days post-EPO using a multiplex immunoassay (U-PLEX Interferon Combo (human), Meso Scale Discovery). LOD indicates the lower limit of detection. Data show mean ± SD of n = 3 independent biological experiments. Statistical analysis of ΔORF2 over WT was performed using unpaired two-tailed Student’s t-test of each time point independently. *: p < 0.05; **: p < 0.01; ns, non-significant. (B) HepG2/C3A cells electroporated with HEV WT or ΔORF2, mixed 1:1 with mock-electroporated cells, were harvested on indicated time points post-EPO for WB analysis. Samples were analyzed for STAT1, pSTAT1, ORF2, and β-actin protein expression. Representative blot of n = 3 independent biological experiments. (TIFF) [file ppat.1013516.s005.tiff]

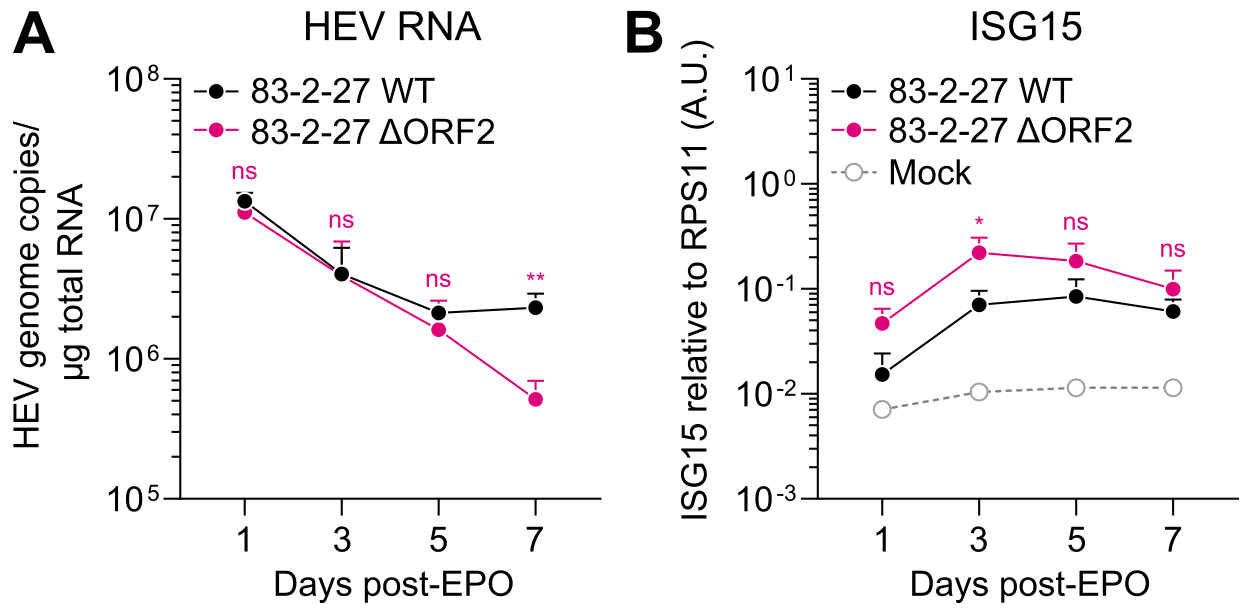

Supplement: S6 Fig — (A) HepG2/C3A cells were electroporated with RNA of the HEV-3 strain 83-2-27 and the derived ΔORF2 mutant. Samples were harvested at indicated time points post-EPO and analyzed by RT-qPCR for HEV RNA genome copies and (B) ISG15 expression relative to the housekeeping gene RPS11 using the 2-ΔCt method. Data show mean ± SD of n = 3 independent biological experiments. Statistical analysis of ΔORF2 over WT was performed using unpaired two-tailed Student’s t-test of each time point independently. *: p < 0.05; **: p < 0.01; ns, non-significant. (TIFF) [file ppat.1013516.s006.tiff]

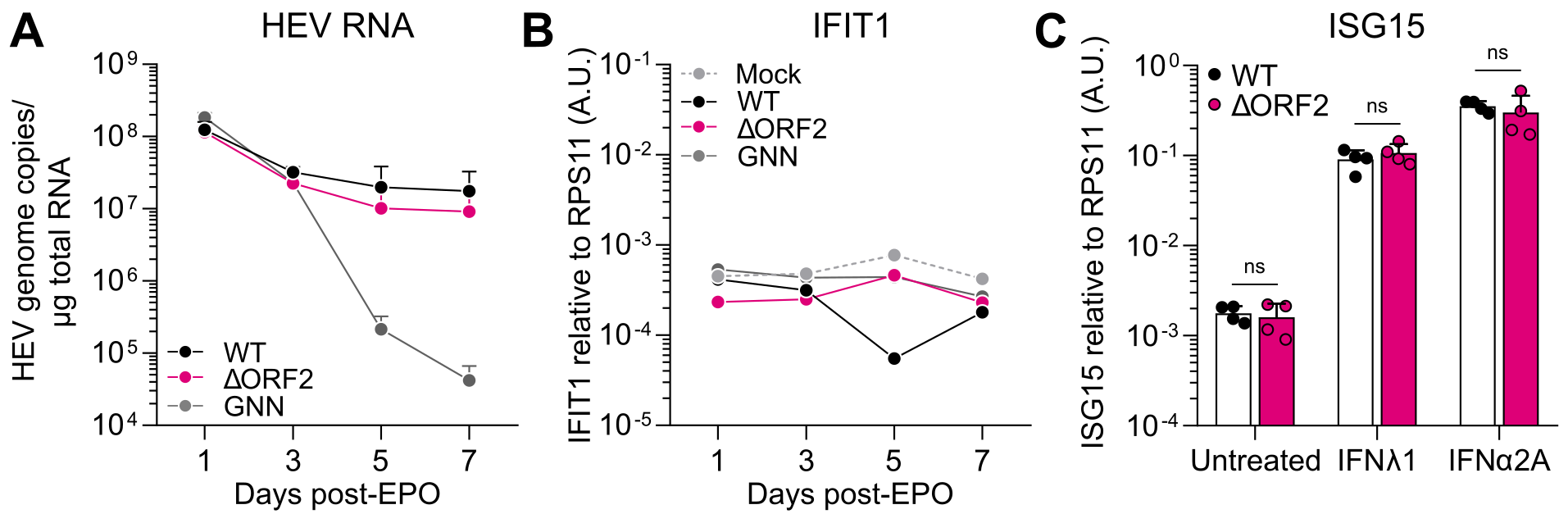

Supplement: S7 Fig — (A) Huh7.5 cells were electroporated with HEV WT, ΔORF2, or replication-incompetent GNN RNA and analyzed at indicated time points post-EPO for HEV RNA by RT-qPCR. Data show mean ± SEM of n = 2 independent biological experiments. (B) Electroporated Huh7.5 cells from (A) were analyzed by RT-qPCR for IFIT1 expression relative to the housekeeping gene RPS11 using the 2-ΔCt method. Data show a single biological experiment. (C) Cell lysates from Fig 2J, treated with IFNλ1 or IFNα2A, were analyzed for ISG15 expression on day 7 post-EPO, relative to the housekeeping gene RPS11 using the 2-ΔCt method. Data show mean ± SD of n = 4 biological repeats from two independent biological experiments. Statistical analysis was performed using two-way ANOVA. ns, non-significant. (TIFF) [file ppat.1013516.s007.tiff]

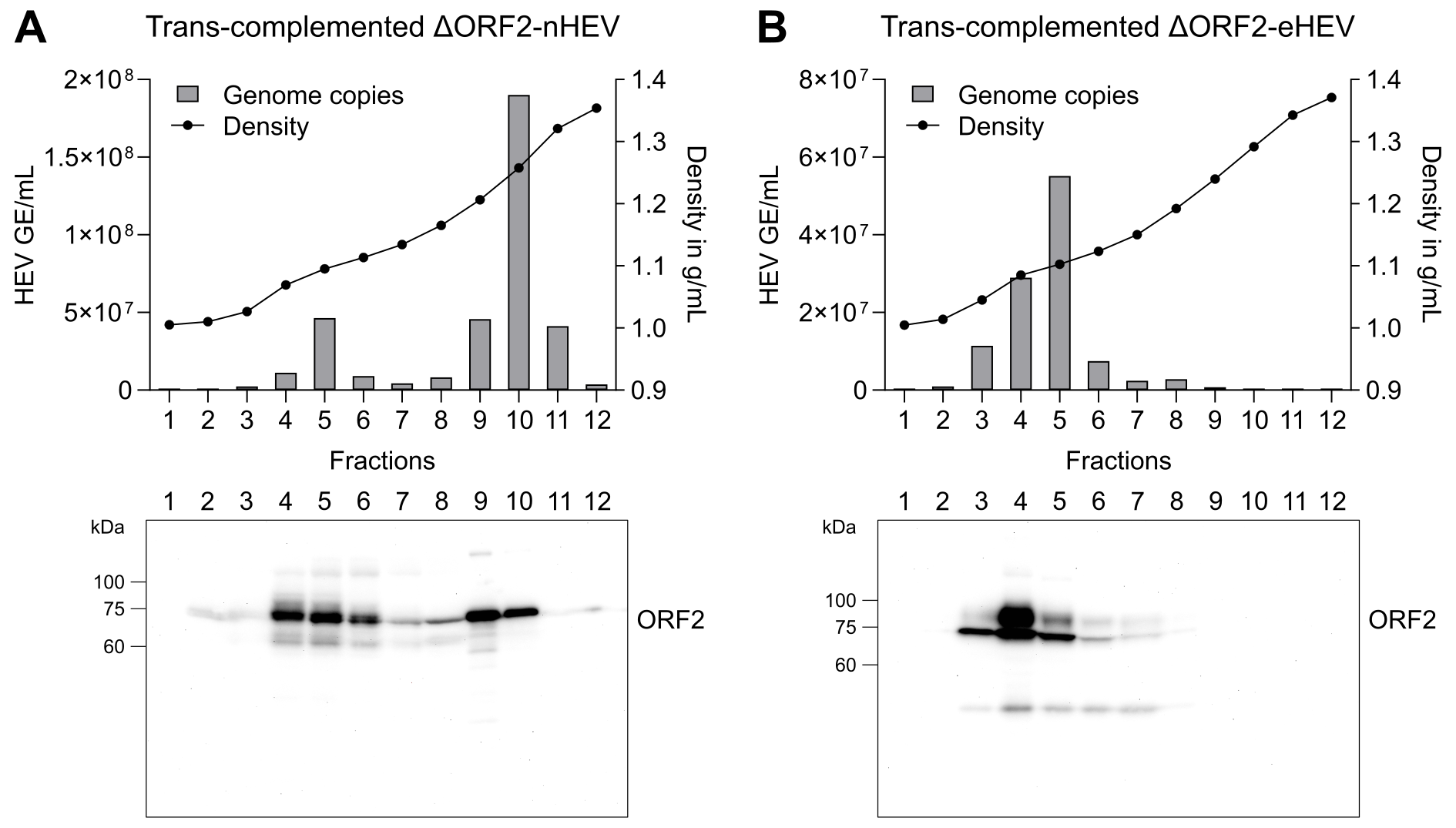

Supplement: S8 Fig — (A) Cell lysates containing nHEV and (B) cell culture supernatants containing eHEV of S10-3/ORF2 cells electroporated with ΔORF2 RNA were purified through density gradient ultracentrifugation. Gradients were harvested in twelve fractions. The buoyant density of each fraction was measured, and HEV GE/mL were determined in each fraction (upper panels). The fractions were further concentrated and analyzed for the presence of ORF2 protein by WB (lower panels). Data show the results of a single ΔORF2 virus production. (TIFF) [file ppat.1013516.s008.tiff]

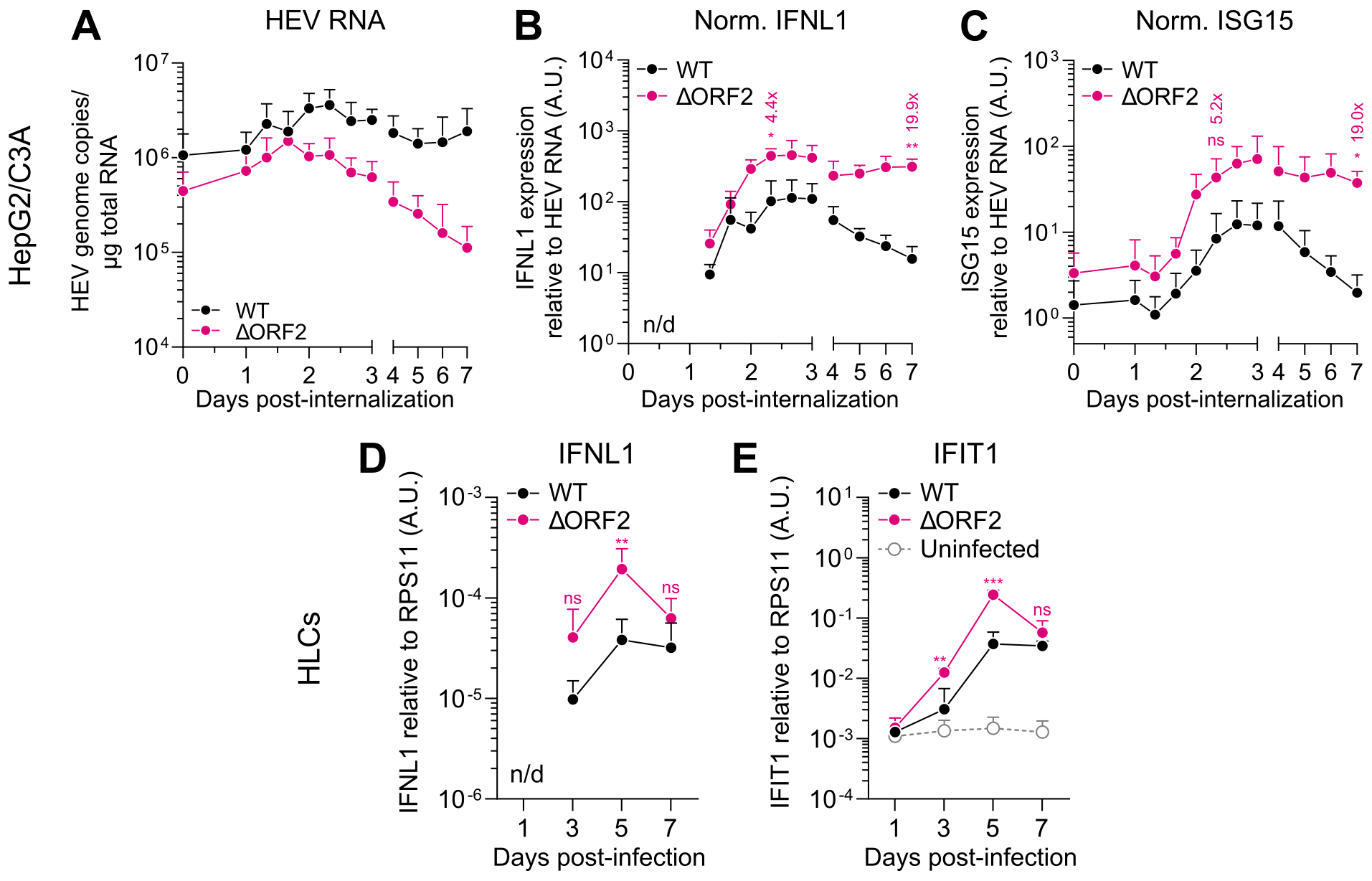

Supplement: S9 Fig — (A) Non-normalized data of Fig 3C: Equal genome copies of HEV WT and ΔORF2 virus particles (30 GE/cell) were bound on HepG2/C3A cells for 2 h at 4°C prior to internalization at 37°C for 8 h, followed by removal of inoculum (= day 0). RT-qPCR was performed at indicated time points post-internalization to determine HEV genome copies. (B) Normalized data of Fig 3D and 3E over HEV RNA: IFNL1 and (C) ISG15 expression relative to the housekeeping gene RPS11 were additionally normalized over HEV RNA using the 2-ΔΔCt method. Statistical analysis of fold changes of ΔORF2 over WT are indicated above the respective time points. Data show mean ± SD of n = 3 independent biological experiments. Statistical analysis was performed using unpaired two-tailed Student’s t-test of the respective days independently. *: p < 0.05; **: p < 0.01; ns, non-significant. A.U., arbitrary units; n/d, not detectable; norm., normalized. (D) Samples from Fig 3G and 3H were analyzed for IFNL1 and (E) IFIT1 expression relative to RPS11 using the 2-ΔCt method. Data show mean ± SD of n = 4 biological replicates of two independent HLC differentiations. Statistical analysis of ΔORF2 over WT was performed using unpaired two-tailed Student’s t-test of each time point independently and is indicated above the respective time points. **: p < 0.01; ***: p < 0.001; ns, non-significant. A.U., arbitrary units; n/d, not detectable. (TIFF) [file ppat.1013516.s009.tiff]

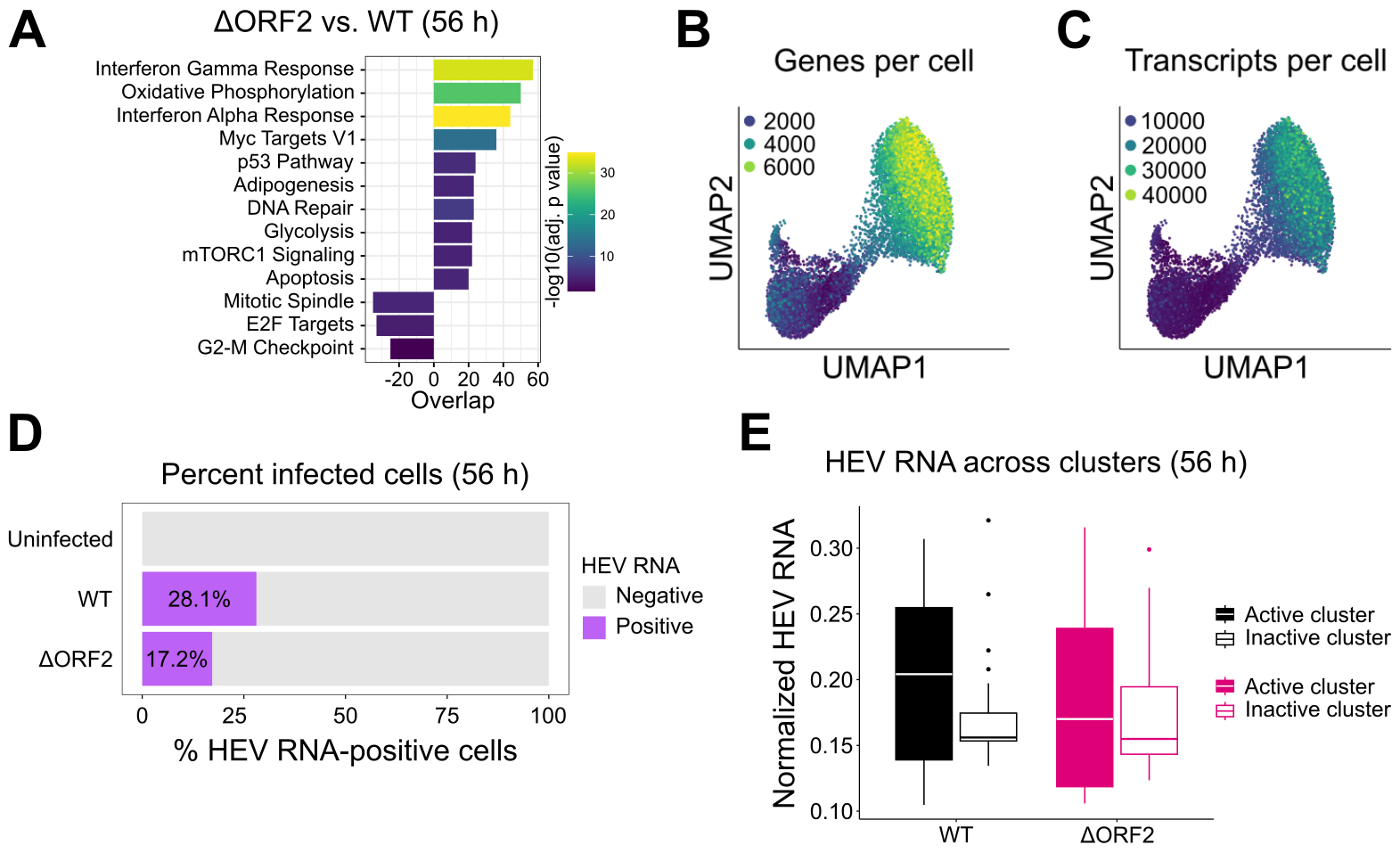

Supplement: S10 Fig — (A) GSEA was performed, comparing the ΔORF2-infected with the WT-infected sample at 56 h post-infection, based on the hallmark gene sets of the Human MSigDB Collections [40]. Positive overlap corresponds to genes upregulated in ΔORF2. (B) UMAP projections of uninfected, WT-, and ΔORF2-infected samples at 56 h post-infection, highlighting the number of different genes detected per cell and (C) the number of transcripts detected per cell in all samples. (D) The percentage of infected cells, defined by at least one detected HEV RNA count, was quantified in uninfected, WT-, and ΔORF2-infected samples at 56 h post-infection. (E) Normalized HEV RNA counts across infected cells in the inactive and active clusters of WT- and ΔORF2-infected samples at 56 h post-infection. (TIFF) [file ppat.1013516.s010.tiff]

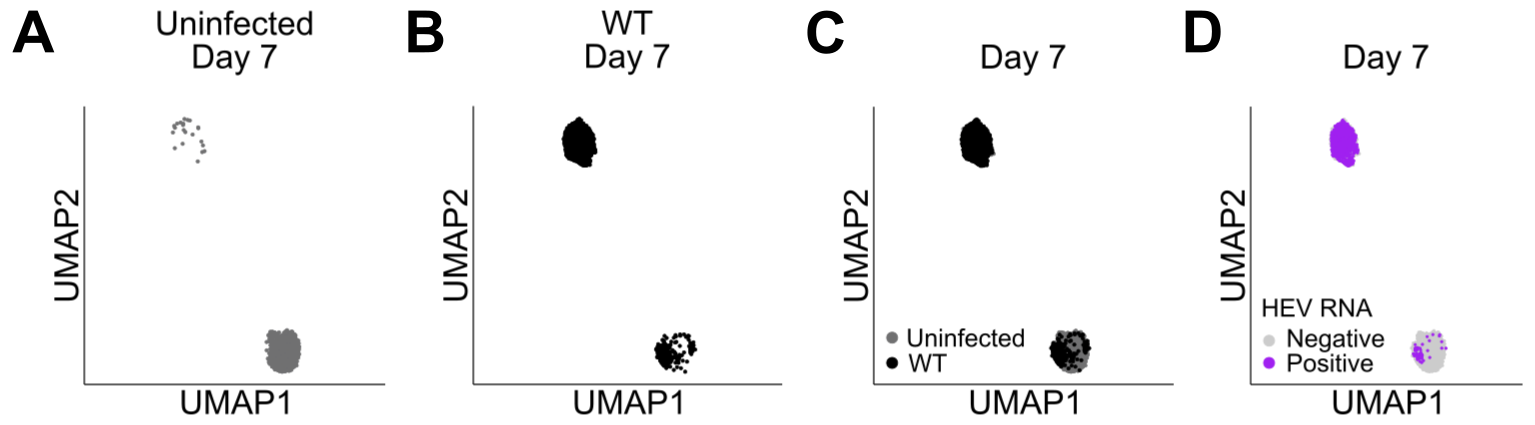

Supplement: S11 Fig — (A) UMAP projections of uninfected and (B) WT-infected HepG2/C3A cells, harvested on day 7 post-infection for scRNA-seq analysis. Cells were clustered based on a list of ~400 ISGs published previously by Schoggins et al. [41]. (C) Combined UMAP projections of uninfected and WT-infected samples, colored by condition. (D) Combined UMAP projection of the uninfected and WT-infected samples on day 7 post-infection with indicated binarized HEV RNA counts in purple. (TIFF) [file ppat.1013516.s011.tiff]
