# Supplementary material for: The hepatitis E virus capsid protein ORF2 counteracts cell-intrinsic antiviral responses to enable persistent replication in cell culture
Source: PLoS Pathog. 2025 Sep 22;21(9):e1013516. doi: 10.1371/journal.ppat.1013516 (PMC12478880; doi:10.1371/journal.ppat.1013516)
Supplement: S1 Table — (DOCX) [file ppat.1013516.s012.docx]

**S1 Table: List of oligonucleotides**

| **Oligonucleotide** | **Target** | **Sequence (5'-3')** | **Ref** |
| --- | --- | --- | --- |
| HEV fw | HEV | GGTGGTTTCTGGGGTGAC | Wu *et al.*[^5^](#_ENREF_5) |
| HEV rv |  | AGGGGTTGGTTGGATGAA | Wu *et al.*[^5^](#_ENREF_5) |
| RPS11 fw | RPS11 | GCCGAGACTATCTGCACTAC | Wu *et al.*[^5^](#_ENREF_5) |
| RPS11 rv |  | ATGTCCAGCCTCAGAACTTC | Wu *et al.*[^5^](#_ENREF_5) |
| IFNL1 fw | IFNL1 | GTGACTTTGGTGCTAGGCTTG | Wu *et al.*[^5^](#_ENREF_5) |
| IFNL1 rv |  | GCCTCAGGTCCCAATTCCC | Wu *et al.*[^5^](#_ENREF_5) |
| ISG15 fw | ISG15 | CTGTTCTGGCTGACCTTCG | Wu *et al.*[^5^](#_ENREF_5) |
| ISG15 rv |  | GGCTTGAGGCCGTACTCC | Wu *et al.*[^5^](#_ENREF_5) |
| GAPDH fw | GAPDH | TCGGAGTCAACGGATTTGGT | Wüst *et al.*[^20^](#_ENREF_20) |
| GAPDH rv |  | TTCCCGTTCTCAGCCTTGAC | Wüst *et al.*[^20^](#_ENREF_20) |
| IFNB1 fw | IFNB1 | CGCCGCATTGACCATCTA | Wüst *et al.*[^20^](#_ENREF_20) |
| IFNB1 rv |  | GACATTAGCCAGGAGGTTCTC | Wüst *et al.*[^20^](#_ENREF_20) |
| TNFAIP3 fw | TNFAIP3 | TCCTCAGGCTTTGTATTTGAGC | Wüst *et al.*[^20^](#_ENREF_20) |
| TNFAIP3 rv |  | TGTGTATCGGTGCATGGTTTTA | Wüst *et al.*[^20^](#_ENREF_20) |
| IFIT1 fw | IFIT1 | GAATAGCCAGATCTCAGAGGAGC | Wüst *et al.*[^20^](#_ENREF_20) |
| IFIT1 rv |  | CCATTTGTACTCATGGTTGCTGT | Wüst *et al.*[^20^](#_ENREF_20) |
| IL6 fw | IL6 | ACTCACCTCTTCAGAACGAATTG | Wüst *et al.*[^20^](#_ENREF_20) |
| IL6 rv |  | CCATCTTTGGAAGGTTCAGGTTG | Wüst *et al.*[^20^](#_ENREF_20) |
| ΔORF2 mutant first start codon fw | HEV ORF2 | TGGGATCACCGTGTGCCCTAG | This study |
| ΔORF2 mutant first start codon rv |  | CTAGGGCACACGGTGATCCCA | This study |
| ΔORF2 mutant second start codon fw | HEV ORF2 | GTTTCTGCCTGTGCTGCCCG | This study |
| ΔORF2 mutant second start codon rv |  | CGGGCAGCACAGGCAGAAAC | This study |
| ΔORF3 mutant fw | HEV ORF3 | CATCGCCCAGCGGATCACCAT | This study |
| ΔORF3 mutant rv |  | ATGGTGATCCGCTGGGCGATG | This study |
| GNN mutant fw | HEV ORF1 | GCCTTTAAGGGTAATAATTCGGTGGT | This study |
| GNN mutant rv |  | ACCACCGAATTATTACCCTTAAAGGC | This study |
| ORF2 2R/2A mutant fw | HEV ORF2 | GTCGTCGTGGGGCGGCCAGCGGCGGTG | Hervouet *et al.*[^21^](#_ENREF_21) |
| ORF2 2R/2A mutant rv |  | GCACCGCCGCTGGCCGCCCCACGACGACG | Hervouet *et al.*[^21^](#_ENREF_21) |
| ORF2 WRD/AAA mutant fw | HEV ORF2 | CTTGGCTCCGCTGCGGCTGCCCAGTCCCAG | This study |
| ORF2 WRD/AAA mutant rv | HEV ORF2 | CTGGGACTGGGCAGCCGCAGCGGAGCCAAG | This study |
