# Supplementary material for: The hepatitis E virus capsid protein ORF2 counteracts cell-intrinsic antiviral responses to enable persistent replication in cell culture
Source: PLoS Pathog. 2025 Sep 22;21(9):e1013516. doi: 10.1371/journal.ppat.1013516 (PMC12478880; doi:10.1371/journal.ppat.1013516)
Supplement: Attachment — Submitted filename: Revision plan_Mehnert et al.pdf [file ppat.1013516.s013.pdf]

Revision Plan

Review COMMONS

Manuscript number: RC-2025-02940
Corresponding author(s): Dao Thi, Viet Loan and Herrmann, Carl

1. General Statements [optional]

We thank the Editors and the Reviewers for their time and constructive criticism, which has allowed us to improve our manuscript. All of our responses are indicated in blue font. Revision Figures for the Reviewers are included just below the response. The line numbers given here refer to those in the revised manuscript, where we have marked the changes in red.

2. Description of the planned revisions

If granted a full revision, we will experimentally address the following major points:

In response to Reviewer 1:

Comment 1a):

1a). The authors conclude from Figure 1 that the HEV ORF2 protein antagonizes both antiviral and inflammatory signaling pathways. The authors comprehensively investigated PRRs-mediated activation of type I interferon by viruses or poly(I:C) through overexpression of MDA5, RIG-I and TLR3. However, they only investigated the impact of ORF2 on host inflammatory response through evaluating the levels of TNFAIP3 RNA in the presence of MDA5 overexpression. It would be informative if the authors also check for NFkB activation/phosphorylation and expression of classical pro-inflammatory cytokines such as IL-1b and IL6.

As suggested by the reviewer, we have also analyzed the impact of ORF2 on TNFAIP3 and IL6 expression in MDA5-overexpressing cells stimulated by poly(I:C) electroporation. Similar to our findings with TNF stimulation we found that the presence of ORF2 reduced the strength of TNFAIP3 and IL6 expression. This data has been added to new Suppl. fig. 2 (mentioned in lines 163–166). In addition, we will analyze TNF-stimulated cells for the expression of other cytokines, such as IL6 and IL-1b. As previously demonstrated, TNFAIP3 is a direct response gene of NF-κB signaling, independent of IRF3 and IFN signaling (Wüst et al., 2021, Cells). Therefore, we do not believe analyzing NF-κB (p65) phosphorylation by Western blot would alter our conclusions.

Comment 2b):

To support their hypothesis, the authors need to further confirm the sudden upregulated antiviral activity on day 5 in electroporated HepG2/C3A cells by testing JAK/STAT phosphorylation and type I interferon secretion.

We agree and will analyze STAT1 phosphorylation by Western blot and type I and III interferon secretion by a multiplex cytokine immunoassay to consolidate the differences in gene expression

levels observed between days 1, 3, 5 and 7 post-electroporation. Preliminary results show that IFNλ1 protein secretion into the cell culture supernatant was greater with ΔORF2 infection compared to WT infection (Revision Figure 1). It also showed that more IFNλ1 protein was secreted on day 5 than on day 7 after infection with the ΔORF2 virus. Type I IFN secretion (IFNα2A, IFNβ) was not detected in these samples (data not shown). We will consolidate these findings by repeating the experiment.

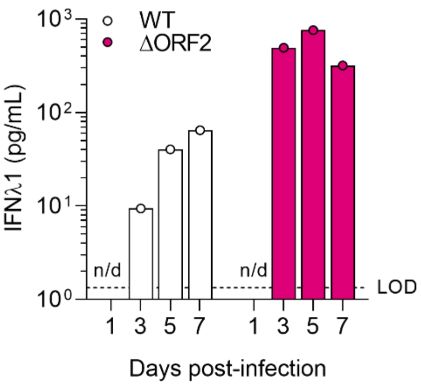

Revision figure 1: ΔORF2 infection results in stronger IFNλ1 secretion than WT infection.
HepG2/C3A cells were infected with HEV WT or ΔORF2. After 8 h, the medium was changed. Supernatants were collected and fresh medium was added at the indicated time points post-infection. IFNλ1 protein levels were measured in the supernatants by multiplex immunoassay (U-PLEX Interferon Combo (human), Meso Scale Discovery). Data represent measurements from a single experiment. LOD, limit of detection; n/d, not detectable

Comment 4a):

The single cell RNAseq data are very informative and revealed two interesting groups of genes. First, the ISGs that are further induced in the cells infected with ORF2-deficient HEV compared to cells infected with WT HEV (Figure 4N) are likely suppressed by ORF2. Second, the ISGs that are uniquely induced in the absence of ORF2 are different from the genes that are uniquely induced by WT HEV (Supplementary Table 2), suggesting that ORF2 may also modulate host gene expression.

We thank the Reviewer for acknowledging this analysis and for pointing out a very interesting aspect. Indeed, it appears that ORF2 not only modulates the cell-intrinsic antiviral response by antagonizing TBK1, as shown in this study, but also has a direct role in modulating host gene expression. A previous study reported that ORF2 harbors an NLS sequence and is likely translocated into the nucleus. The nuclear form of ORF2 was then reported to influence the cytokine response in infected cells (Suppl Figure 6, Hervouet et al.; 2022, PLoS Pathogens). While we have not yet confirmed these observations, many viral proteins are able to translocate into the nucleus and thereby exert different effects on host transcription. These proteins can modulate gene expression by targeting transcriptional machinery or by altering chromatin states (reviewed in Liu et al.; 2020, Cell). One prominent example is the Epstein-Barr virus nuclear antigen 2 (EBNA2) which interacts with histone acetyltransferases (HATs), transcription factors, and the basal transcription machinery to control host gene expression (Yan et al.; 2021, Communications Biology). As a result of this very constructive comment, we have already expanded on this aspect in our discussion section (lines 461-467) and now explicitly discuss the potential of ORF2 not only to modulate host gene expression by antagonizing TBK1, but also to

affect it directly. We and others need to extend these observations and better characterize the impact of nuclear ORF2 translocation on host gene expression.

As part of a full revision, we will revisit our scRNA-seq data to more closely investigate the differences in the differential gene expression between infected cells and bystanders in both WT and ΔORF2 infections. We are generating scatter plots that show a direct comparison of the differential expression of ISGs and other genes between WT and ΔORF2 infections, as shown in Revision figure 2. We are already in the process of performing gene set enrichment analysis on the genes specifically upregulated in WT infection compared to ΔORF2 infection, which are highlighted in green in Revision figure 2. We are also making use of RT-qPCR analysis to confirm the specific upregulation of a subset of these genes in WT infection. We believe this analysis will reveal more detailed aspects of the differences in the transcriptional responses of WT and ΔORF2 than our current Venn diagram (Fig. 4L) shows.

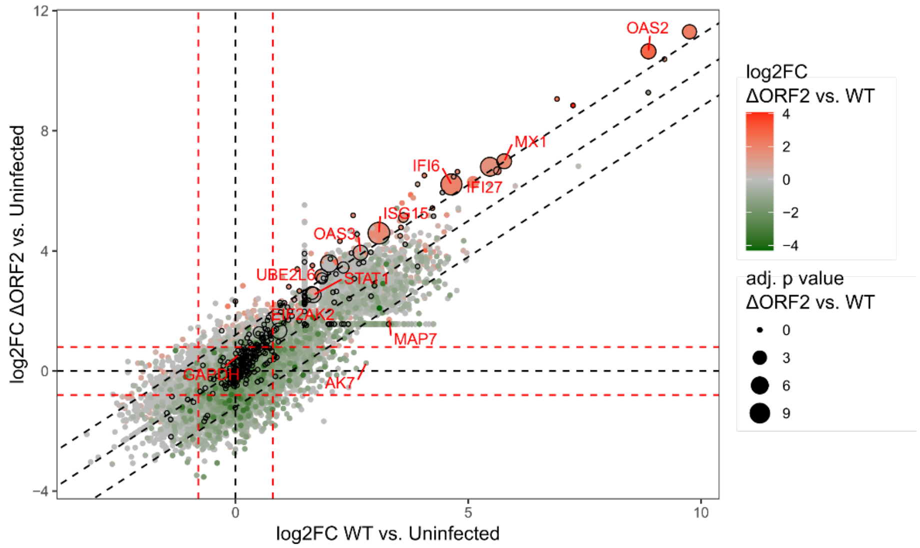

Revision figure 2: Comparison of differential gene expression between WT and ΔORF2. Scatter plot showing log2(fold change) (log2FC) of all genes, comparing WT-infected cells vs. uninfected sample on the x-axis and ΔORF2-infected cells vs. uninfected sample on the y-axis. Genes colored in red have higher expression in WT compared to ΔORF2, while genes colored in green have higher expression in ΔORF2 compared to WT. Dot sizes represent the adjusted (adj.) p value of the WT vs. ΔORF2 comparison. Dots highlighted by a black border are part of the ISG gene list according to Schoggins et al.; 2011, Nature.

Comment 4c):

Related to that, are there other gene expression changes beyond ISG signatures which would suggest that ORF2 can regulate host gene expression? Figure 4A-C only shows comparisons for WT or ORF2-deficient vs. uninfected cells. The authors can perform GO and KEGG analyses to see if certain biological processes/pathways are enriched among the WT vs ORF2-deficient HEV induced genes. Further characterization of these genes (ISGs or not) would shed light on the novel roles of ORF2 in both immune antagonism and gene regulation and greatly increase the significance of the study

This is an excellent point, and we thank the Reviewer for bringing it to our attention. Accordingly, we have performed a gene set enrichment analysis of ΔORF2 vs. WT samples at the 56 h time point and added it to the revised Supplementary information as new Suppl. fig. 7A (mentioned in lines 367-369). This analysis revealed that differences in the ISG response and metabolic changes are most significantly affected by ΔORF2 infection compared to WT infection. As detailed in our response above (comment 4a), we are currently revisiting our scRNA-seq data to more closely investigate the differences in the differential gene expression between infected cells and bystanders in both WT and ΔORF2 infections, especially since many viruses are known to interfere with oxidative phosphorylation pathways (reviewed by Moreno-Altamirano et al.; 2019, Front Cell Infect Microbiol).

In response to Reviewer 2:

Comment 1:

Did the authors study only the P6 HEV genome? Have they done anything comparative with the other strain to understand if the proposed mechanism is not the strain specific?

As shown in Figure 1, we found that both ORF2 from HEV-1 and HEV-3 can antagonize antiviral and inflammatory signaling pathways to a comparable extent. We will complement this analysis by generating a ΔORF2 Sar-55 HEV-1 strain and comparing its replication and antiviral response gene induction capacity with WT Sar-55 HEV-1 in hESC-derived HLCs (also in response to Reviewer 3).

In response to Reviewer 3:

Comment 1:

The researchers used p6, a cell-adapted clone, which was isolated form a chronic HEV patient. As previous studies suggested, p6 may behave differently than wild-type strains. Did the authors tried other HEV strains, as they used ips-induced model that was reported supportive to wild-type HEV?

As also outlined in our response to Reviewer 2, we found that both ORF2 from HEV-1 and HEV-3 can antagonize antiviral and inflammatory signaling pathways to a comparable extent (Figure 1). We will complement this analysis by generating a ΔORF2 Sar-55 HEV-1 strain and comparing its replication and antiviral response gene induction capacity with WT Sar-55 HEV-1 in hESC-derived HLCs.

Minor comment 1:

Figure 3A, this is an elegant design. More data may provide for the validation of the formation of the virions.

In order to demonstrate the formation of infectious particles, we will separate naked and quasi-enveloped HEV particles by density gradient ultracentrifugation and detect the ORF2 capsid by Western blot analysis and the HEV genomes by RT-qPCR.

3. Description of the revisions that have already been incorporated in the transferred manuscript

In response to Reviewer 1:

Comment 1b:

Interestingly, changes in IFNB secretion after ORF2 overexpression appear more dramatic compared to changes in IFNB1 RNA levels (compare Figure 1A-C with Supplementary Figure 1A and C). Are the IFN-beta protein expression changes statistically significant in Supplementary Figure 1?

The changes in protein expression (absolute values) are likely to appear more dramatic due to the linear scale as opposed to the logarithmic scale used in Figure 1A-C (relative values normalised to housekeeping gene expression). We have calculated the fold reduction in gene expression in cells expressing HEV-3 ORF2 and HEV-1 ORF2 relative to cells expressing GFP and added this information to both figures (line 966 and Supplementary information, lines 144-145) and to the Results section (line 152), showing that the differences are similar. In addition, we have added information on the statistical differences in new Suppl. fig. 1C-D. As shown in this updated figure, the changes in IFNβ protein secretion are also significant when ORF2 is ectopically expressed (lines 154-156).

Comment 2a):

Changes in the IFN response do not always translate into changes in the viral RNA levels. In Figure 2B-D, the authors attributed the higher induction of IFNL1 and ISG15 on day 5 to the absence of ORF2 inhibition. However, the expression of these two genes drops to the same levels as the ones in WT viral RNA-electroporated cells on day 7, which is strange as ORF2-deficient viral RNA levels continue to be inhibited on day 7. This is different from the stem cell derived hepatocytes infected with the trans-complementation viruses in Figure 3G-H where there are significant differences in ISG15 levels between WT and ORF2-deficient virus infected cells on both days 5 and 7.

Indeed, we believe that by day 7, viral replication, regardless of which virus (WT or ΔORF2), induces a cell-intrinsic antiviral response that equilibrates to a level where both replication and the response can coexist. In Figure 2B-D, ΔORF2 replication is dampened to a level where it

induces an apparently similar response to WT replication. However, as shown in Suppl. fig. 4A-B, when the response is normalized to the number of replicating ΔORF2 genomes, the induced response is relatively higher due to the absence of the ORF2-mediated antagonism. We have added a statement in the revised manuscript for clarification (lines 219-221).
Regarding the apparent discrepancy observed in HLCs, we have also analyzed other genes besides ISG15, namely IFNL1 and IFIT1, and found that these genes are induced to a similar extent by both WT and ΔORF2 infection of HLCs on day 7. We are now showing these data in the new Suppl. fig. 6D-E. As we show ISG15 as a representative ISG throughout the manuscript, we would prefer to keep this panel in the main figures. However, we have explicitly added a statement in the revised manuscript stating that there are no apparent differences in the induction of IFIT1 and IFNL1 levels on day 7 between WT and ΔORF2 infection (lines 334-337).

Comment 3a):

The authors used different hepatocyte systems coupled with viral RNA electroporation or trans-complementation virus infection to investigate ORF2-mediated interference of the IFN pathway, which is highly complementary. However, while the electroporation of viral RNA into HepG2/C3A (Figure 2B-D) and infection of stem cell-derived hepatocytes with trans-complementation viruses (Figure 3F-H) result in similar upregulation of ISG expression on day 5, that wasn't observed in HepG2/C3A cells infected with trans-complementation viruses (Figure 3C-E) on day 5. The authors need to discuss the discrepancy among these different systems.

As the Reviewer points out correctly, due to the different routes of viral genome delivery (e.g., electroporation vs. authentic infection with full-length virus vs. synchronized and non-synchronized infection with trans-complemented ΔORF2 virus) and the different cellular backgrounds used (HepG2/C3A vs. HLCs), direct comparisons, especially with respect to temporal dynamics, are difficult to make. Therefore, we are careful not to make them in our manuscript.
We used electroporation to deliver WT, ΔORF2 or ΔORF3 genomes into cells, bypassing the cell entry steps. This allowed us to directly compare and determine the viral protein involved in enabling efficient viral replication. Due to their inherently different roles in progeny packaging (ORF2) and progeny secretion (ORF3), a comparison based on trans-complemented particles would be rather difficult. We have made this clearer in the Results section (lines 205-209).
Having developed a specific assay that allowed us to study the dynamics of HEV cell entry, we previously discovered that the steps leading to successful delivery of the genome into the cytoplasm are a rather slow process for HEV compared to other viruses (Fu et al.; 2023, BioRxiv, in press at Nat Commun). In fact, the majority of infectious naked HEV particles do not deliver their genome to cells until 12 hours later (Figure 3, Fu et al.; 2023, BioRxiv, in press at Nat Commun). Since electroporation bypasses this step, it is not possible to compare the kinetics of replication and the cell-intrinsic antiviral response following electroporated versus infection-mediated delivery of the genome.
In addition, we believe that the requirements for the initiation of successful replication are likely facilitated in HepG2/C3A cells compared to HLCs. As already observed in our previous publication

(Figure 3 in Wu & Dao Thi et al., 2018, Gastroenterology), the onset of replication occurs on day 3 post-infection in HLCs, which is perfectly consistent with the observations in the present study by Mehnert et al. (Figure 3G, H). In HepG2/C3A cells, the onset of replication occurs earlier, already from day 2, which is in agreement with the study by Yin et al.; 2018, PLoS Pathogens (Figure 1B). Therefore, in the present study, we strictly avoid comparing the kinetics of replication and cell-intrinsic antiviral response over time between the different systems used. Why replication occurs earlier in HepG2/C3A may be due to the presence of a facilitating host factor that is absent in HLCs or the presence of a restricting host factor (such as an ISG) that is present in HLCs. The identification of such factors should be addressed in future studies and we have added this aspect to the discussion section. We have also added a more detailed explanation of the difference in the culture systems used (hepatoma cell line vs. hESC-derived hepatocyte-like cells) to make this difference clearer (lines 496-506). However, we appreciate the Reviewers' acknowledgement that these systems are highly complementary, although they do not allow direct comparisons of viral replication kinetics.

Comment 3b):
Since the ORF2-deficient trans-complementation virus still brings in ORF2 proteins from the producer cells but cannot generate new ORF2 proteins, do ORF2 proteins from these two different sources have different functions in different hepatocyte systems? In addition, other than the data points that are shown to be not significantly different in Figure 3D-E, are any of the other data points significantly different?

This is a good point. In our recent publications, we have found that the activity of lysosomal cathepsins appears to be critical for successful HEV entry. Indeed, we discovered that cathepsin can directly lead to cleavage of the viral capsid and that cathepsin inhibitors prevent HEV infection (Figure 5 and 6F, Klöhn et al.; 2024, Hepatology). In addition, using an antibody against the capsid in combination with detection of the viral genome by RNA-FISH, we further confirmed that the capsid is likely degraded in the lysosome upon entry (Figure 3B-E, Fu et al.; 2023, BioRxiv, in press at Nat Commun). Therefore, it is unlikely that the ORF2 protein itself is being delivered to the infected cell from the incoming trans-complemented particles or from the WT particles. Therefore, we do not expect any differences in the function or activity of either ectopic ORF2 (expressed only in the producer cells) or ORF2 expressed by viral replication.
In Figure 3C, the replication between WT and ΔORF2 is significantly different following the bottleneck at 56 h post-infection. Although ΔORF2 replicates at much lower levels than WT (Fig. 3C and Suppl. fig. 6A), it leads to a similar induction of IFNL1 and ISG15 (Fig. 3D, E). When the response is normalized to viral replication, as shown in Suppl. fig. 6B-C, it becomes clearer that the ΔORF2 virus induces a significantly higher response per replicating genome than the WT virus. We have made this clearer in the revised Result section of the manuscript (lines 318-323). We do not want to mislead the reader by only presenting the normalized data, which could raise the impression that the ΔORF2 virus induces an overall stronger antiviral response than the WT virus in HepG2/C3A cells. Therefore, we prefer to keep the non-normalized representation of the data in the main figures and the normalized data in the supplementary figures. We made it clearer in the text that the normalized data can be found in the supplements.

Comment 5:

In Supplementary Figure 3F-H, the authors used BX795 to inhibit TBK1 (a target of ORF2) and found decreases in IFNL1 and ISG15 expression whether cells are electroporated with WT, ORF2-deficient, or ORF3-deficient viral RNA. However, this does not correlate with the data in Figure 2E-G where TBK1 inhibition results in significant differences in viral RNA levels only in the absence of ORF2 or ORF3. These results would suggest that the effects of TBK1 inhibition on viral RNA levels is independent of changes in the IFN/ISG expression levels.

Indeed, the TBKi treatment has a stronger effect on ΔORF2 than on WT or ΔORF3 replication which we believe aligns perfectly with our discovery that ORF2, on the one hand, antagonizes the cell-intrinsic antiviral response (Fig. 1), and on the other hand, shields viral replication from the action of antiviral effectors (Fig. 2J). In contrast, WT replication is less vulnerable to the action of antiviral effectors and is therefore not significantly enhanced when exogenously blocking TBK1, even though the treatment leads to a decrease in ISG expression. Therefore, this observation introduces for the first time the additional protective function of ORF2 that is also confirmed in Fig. 2J.
The data shown in Suppl. Figure 3F-H served as a control to show the potency of TBK1 inhibition to exogenously reduce the virus-induced ISG response in cells electroporated with WT and mutant HEV. Therefore, the same cell lysates were used for Suppl. Fig 3F-H and Figure 2E-G, and we have added this information explicitly to the Figure legend (lines 195-199 in Supplementary Information). We have further added information on the fold changes between DMSO and TBKi-treated cells in Suppl. fig. 4F-H, which shows that on day 5 post-EPO, ΔORF2 virus induces a stronger response than WT or ΔORF3 virus in the absence of TBKi treatment, which can also be seen in Fig. 2C, D. It further highlights that the TBK1 inhibitor treatment reduces the antiviral response to a similar level for WT, ΔORF2 and ΔORF3. Overall, this substantiates our observation that inhibition of the antiviral response enhances ΔORF2 replication more significantly than WT or ΔORF3 replication, suggesting that ΔORF2 replication is more vulnerable to the action of ISGs. We have adapted the revised Result section to make these observations clearer (lines 239-246 and lines 265-266).

In response to Reviewer 2:

Minor comments:

Line 69, 71 - I have never seen in any paper including reference in this way!

We removed the respective citation style, as suggested, to make the manuscript more appealing to the reader.

Line 72 and 73 - missing reference!

We added the missing reference.

Line 92, 93 - missing reference!

We added the missing reference.

Line 95 to 99 - missing references!

We added the missing reference.

Major comment 2:

Can the authors explain why we do not see any band in the Fig. 1F B-actin?

We do not expect to detect β-actin in our IP samples as no specific interaction between ORF2 and β-actin has been reported so far. Therefore, the lack of a β-actin band in our sample after the IP highlights the reliability and accuracy of the assay, and moreover, it underlines the specificity of the interaction between ORF2 and TBK1.

In response to Reviewer 3:

Comment 2:

Figure 1F, ORF2 can interact with TBK1 as showed. But the prediction from Alphafold is weak. Also, could the author more evidence than the co-IP?

AlphaFold has recently emerged as a very powerful and important tool for predicting protein-protein interactions. However, our collaborator Dr. Thibault Tubiana, who performed the predictions for us, has found that AlphaFold does not perform well when predicting interactions with large proteins. Therefore, we only modeled the potential interactions between TBK1 and individual segments of the ORF2 protein (N-terminus, S-, M-, or P-domain). We could not identify a reliable interaction, but the low PAE scores (as shown in Supplementary Fig. 3E) do not fully rule out that the full-length ORF2 protein still interacts with the TBK1 dimer. As we discuss in our manuscript (lines 526-539), it is also possible that the interaction occurs with the unstructured ORF2 N-terminus, and thus modeling the interaction with AlphaFold is likely to be challenging.
In order to confirm ORF2 and TKB1 interaction aside from the IP shown in Fig. 1F, we also performed co-localization studies based on immunofluorescence staining and confocal microscopy. As shown in Revision Figure 3, we observed a strong co-localization between ectopically expressed TBK1 and large ORF2-positive structures, which further confirms their interaction. It should be noted that for this analysis, we had to detect V5-tagged TBK1 due to the lack of appropriate antibodies capable of detecting endogenous TBK1.

However, we believe that the stringent conditions of the co-IP presented in our manuscript more convincingly supports a direct interaction between ORF2 and TBK1 than co-localization by immunofluorescence staining. The interaction between ORF2 and TBK1 has already been published previously (Lin et al.; 2019, Viruses), and our data presented here is in agreement with this study. Furthermore, we are still in the process of studying and characterizing the large ORF2-derived structures observed by immunofluorescence as seen in the Revision Figure 3. As this is part of current and future work in our laboratory, we prefer not to show these images in our manuscript. However, we believe they further consolidate the interaction between HEV ORF2 and TBK1.

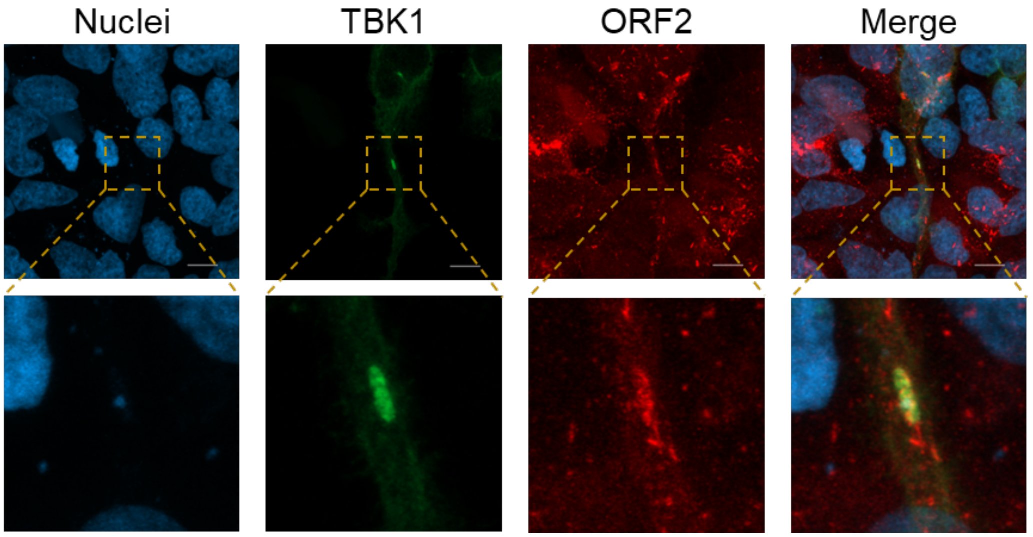

Revision Figure 3: ORF2 co-localizes with TBK1.
S10-3 cells were electroporated with *in vitro* transcribed HEV WT RNA. After 4 days, the cells were transfected with a plasmid encoding TBK1-V5. On day 2 post-transfection, the cells were fixed with 2% PFA. The cells were treated with cold methanol for 20 min, blocked and permeabilized with 10% goat serum/1% BSA/0.1% Triton X-100 in PBS for 1 h, and incubated with primary antibodies against ORF2 (1:400, Merck, MAB8002) and V5 tag (1:1000, Cell Signaling Technology, #13202) at 4 ℃ overnight. The cells were incubated with fluorophore-conjugated secondary antibodies (1:1000) and Hoechst 33342 (1:1000) for 1 h at room temperature. Images were taken with a Zeiss LSM900 Airyscan 2 microscope. Z stacks have a 0.3 μm interval. Maximum projection images are shown. Scale bar, 10 μm.

Comment 3:

Figure 2C and 2D, at 5 dpi, one can observed a stronger antiviral response, but at 7 dpi, no obvious difference was observed. Could the authors comment on this?

Indeed, and as clarified in our response to Reviewer 1 (#2a), we believe that around day 5 post-EPO, ΔORF2 induces a strong response that subsequently leads to a decline in viral replication. By day 7 post-EPO, similar to WT, ΔORF2 replication and the antiviral response equilibrate to a level where both can coexist. However, ΔORF2 replication is more dampened compared to WT replication due to the lack of the TBK1 antagonism, protection from antiviral effectors, and modulation of the host response. We have clarified this in the updated Results section (lines 219-221).

Comment 4:

Figure 2H and 2I, detailed description of how the authors measured the positive cells should be provided. Did the authors selected whole plate of cells for counting? As showed in Figure 2H, the signals of IF were stronger at 5 and 7 dpi when compared at 3 dpi, but why the proportion of positive cells was reduced in Figure 2I?

We apologize for the confusion. The images shown in Figure 2H and I are not based on IF staining but on RNA-FISH, a method that is based on fluorescent probes that allow the detection of

Review COMMONS

individual HEV genomes (Fu et al., 2023, BioRxiv, in press at Nat Commun). Due to the absence of the viral proteins ORF2 or ORF3, we decided to quantify infected cells based on the detection of viral genomes, allowing the direct comparison between different viruses, including the WT virus. Therefore, an increase in signal per cell indicates an increase in replication per cell over time, which is why the signals are stronger at days 5 and 7 compared to day 3 (Fig. 2H). The observation that the number of positive cells slightly decreases over time for WT and ΔORF3 indicates that the antiviral response is likely able to limit not only the replication per cell but also affects the percentage of infected cells. For the ΔORF2 virus, both the replication per cell and the number of replicating cells decrease over time, highlighting the critical role of ORF2 in providing HEV with the ability to persistently replicate in immunocompetent cells. We have made the use of RNA-FISH for this analysis in the Results section more explicit in the revised manuscript (lines 247-259).

In addition, we have added to the updated Figure legend of Figure 2 (line 1001) and in the Supplementary Methods ("RNA fluorescence in situ hybridization and quantification", lines 40-41) the information that we used five randomly selected fields of view per condition and biological replicate for the quantification.

Comment 5:

The study emphasized the function of ORF2 on HEV "persistence". However, this cannot be fully supported by cell models. In future, study on chronic HEV infection animal models may be conducted.

Recent discoveries of inborn errors of immunity have taught us that the cell-intrinsic response is critical in limiting pathogen infection. In the present manuscript, we have investigated how HEV achieves persistent viral replication in immunocompetent in vitro cell culture models. We believe we have provided strong evidence that in the absence of the capsid protein ORF2, HEV replication cannot be sustained due to the efficient cell-intrinsic antiviral response in immunocompetent cells. Since we are fully aware that cell culture models based on monocultures of hepatocytes are not sufficient to identify the determinants that lead to viral persistence and chronicity *in vivo,* we refrain from making any such claims. Instead, we and others strongly believe that infected hepatocytes affect professional immune cells, which in turn are critical for efficient B- and T-cell responses *in vivo*. Therefore, a detailed understanding of the viral interference with the cell-intrinsic antiviral response constitutes an important basis for more complex studies. We thus propose to consolidate our findings in more complex models, e.g. based on co-culture of infected hepatocytes and innate immune cells, for example pDCs. We have now explicitly added the limitations of our study together with the notion that in the future, our findings on ORF2 should also be complemented and consolidated in immunocompetent animal models (lines 562-568).

Comment 6:

The authors study ORF2 in whole. It will be of benefit to the readers that the authors could specified the function of secreted ORF2 and ORF2 capsid in the current study.

This is a very interesting point. Indeed, we have previously tested the ability of the different isoforms of ORF2, namely intracellular ORF2i and glycosylated ORFg, to enable viral replication in immunocompetent cells. For this purpose, we mutated the respective start codon in the full-length genome according to the strategy described by Yin et al.; 2018, PNAS (Revision Figure 4A). Removal of the glycosylated form allowed the ORF2i virus to replicate in the presence of a dampened antiviral response similar to WT. This suggested that the cytoplasmic isoform ORF2i is sufficient to inhibit TBK1 and shield viral replication from antiviral effectors.
However, when we attempted to remove the intracellular form and only retained ORF2g, we observed an intermediate phenotype. The ORF2g virus replicated to lower levels compared to the ORF2i and the WT virus, but not as low as the ΔORF2 virus (Revision Figure 4B). Similarly, it induces a stronger response than ORF2i and WT virus, but not as strong as the ΔORF2 virus (Revision Figure 4C, D). Ongoing work in our lab has shown that it is very likely that some of the ORF2g isoform is retained in the cytoplasm despite expression of the signal peptide. Since we still need to consolidate these data and it remains a matter of debate in the field how the maturation and localisation of the different ORF2 isoforms is regulated (Yin *et al.* 2018, PNAS; Hervouet *et al.* 2022, PLoS Pathogens), we prefer not to show these data in the manuscript. However, we acknowledge that this should be conclusively studied in the future, and we have included this aspect in the Discussion section of the revised manuscript (lines 513-517).

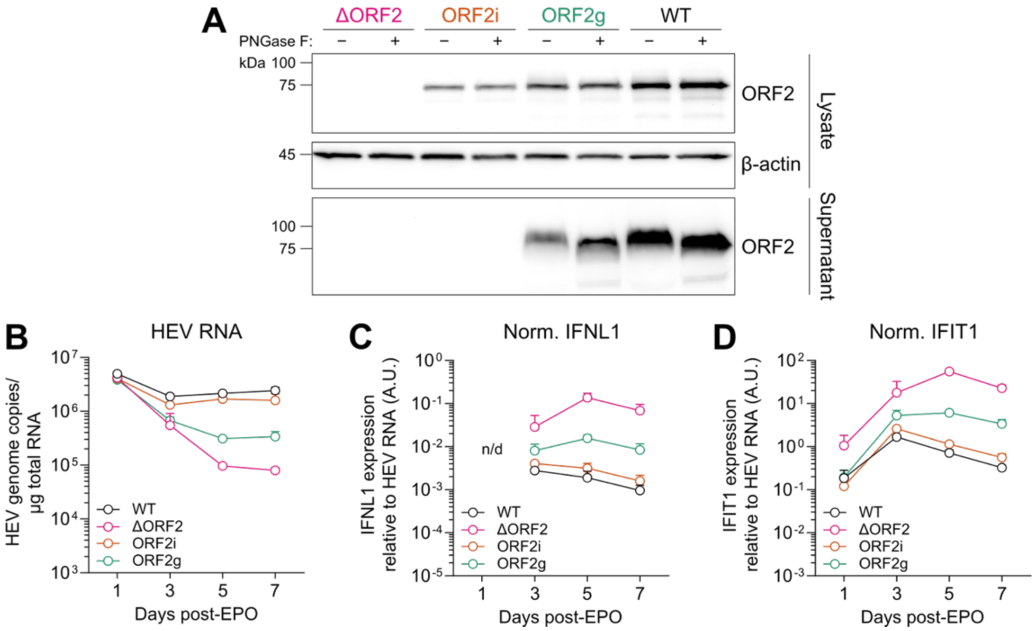

Revision figure 4: The ORF2i isoform rescues the ΔORF2 phenotype.

(A) HepG2/C3A cells were electroporated with IVT RNA of HEV WT, ΔORF2, and full-length HEV mutants expressing only ORF2i or ORF2g. On day 7 post-EPO, cell lysates and supernatants were harvested, treated with PNGase F to remove N-glycosylations and then analyzed by Western blot for protein expression of ORF2 and β-actin. Representative blot of n = 2 independent biological experiments. (B) HepG2/C3A cells electroporated as described in (A) were analyzed for HEV RNA by RT-qPCR. (C) *IFNL1* and (D) *IFIT1* expression was analyzed relative to *RPS11* and normalized to HEV RNA using the $2^{-\Delta\Delta Ct}$ method. Data show mean ± SEM of n = 2 independent biological experiments. A.U., arbitrary units; n/d, not detectable; norm., normalized.

Minor comment 2:

Figure 1, data should be provided for the successful expression of HEV-1 or HEV-3 ORF2, and ORF3.

We have analyzed the A549-derived cell lines for successful expression of HEV-1 and HEV-3 ORF2 as well as HEV-3 ORF3. We have added the respective Western blot analysis as panel A to the new Suppl. fig. 1 (mentioned in lines 146-147).

Minor comment 3:

line 219, the current evidence that supported this statement is weak, especially for ORF2.

We have adjusted and toned down our interpretation in the manuscript, acknowledging that we can only make an assumption about the effect of secondary virus spread in our system (lines 229-232).

Minor comment 4:

Suppl Figure 3F-3H, statistical analysis is needed

We have added the statistical analysis to Suppl. fig. 4F-H, also as described in our response to comment 5 of Reviewer #1.

Minor comment 5:

Suppl Figure 3F-3H, it seems that when no treatment was admistrated, the level of ISG15 in ΔORF2 group was higher than those of the WT and ΔORF3 group. Could the authors comment?

Indeed, in absence of the TBKi treatment, electroporation with the ΔORF2 genome resulted in a stronger induction of ISG15 on day 5 post-EPO compared to WT and ΔORF3. The data in Suppl. fig. 4F-H corresponds to the data shown in Fig. 2B-G, where this phenotype is already visible. We discuss in our manuscript that the lack of TBK1 antagonism mediated by ORF2 probably causes the stronger induction of the antiviral response in the ΔORF2 mutant. We have added a statement in lines 221-223 to make this observation clearer to the reader.

Minor comment 6:

Figure 3D and 3E, the starting time of the detection is not aligned.

We apologize for the confusion. While ISG15 expression is detectable at basal levels and already slightly increased 24 h post-internalization, IFNL1 expression is not detectable at basal levels. We only detected IFNL1 mRNA 32 h post-internalization. For clarification, we added "n/d" (not detectable) to the IFNL1 graph prior to the 32-hour time point to better align the graphs.

Minor comment 7:

Figure 3F, scale bar is missing.

We have added scale bars to all images in Fig. 2 and Fig. 3.

Minor comment 8:

In M&M, statistical method should be provided with more details and cover all the experiments used.

As different statistical tests had to be used for different experiments in our study, depending on the type of comparison to be made, we specified the statistical analysis used for each panel in the figure legends. We prefer to keep it this way as we believe that otherwise it will be difficult to assign the correct statistical analysis. A note is given in the Methods section clarifying that information on statistical tests and p-values is given in the respective Figure legends (lines 706-707).

4. Description of analyses that authors prefer not to carry out

In response to Reviewer 1:

Comment 4b:

The authors can further characterize these two groups of ISGs by performing gene knockdown or knockout and investigating whether ORF2 directly interacts with these ISG products to determine the functional consequences of their upregulation.

This is a very good suggestion. However, we would prefer not to perform this experiment at this time as our collaborators have already performed an ISG screen based on the Schoggins library (Schoggins et al.; 2011, Nature) and will be publishing these results soon. In addition, we believe that it is rather unlikely that ORF2 can modulate gene expression in a targeted manner by antagonizing TBK1 or by affecting transcription in the nucleus. In addition, since the amount of additional work (siRNA screen, co-IPs, etc.) would heavily delay the publication of our manuscript, we would prefer not conducting this analysis at this stage. However, we fully agree that this is an interesting aspect that should be followed up on in the future, once we have a better understanding of how the nuclear form of ORF2 alters host transcription. We have added this aspect to the Discussion section, as already detailed above (lines 461-467).
