# Supplementary material for: The hepatitis E virus capsid protein ORF2 counteracts cell-intrinsic antiviral responses to enable persistent replication in cell culture
Source: PLoS Pathog. 2025 Sep 22;21(9):e1013516. doi: 10.1371/journal.ppat.1013516 (PMC12478880; doi:10.1371/journal.ppat.1013516)
Supplement: Attachment — Submitted filename: Rebuttal letter_Mehnert et al.docx [file ppat.1013516.s014.docx]

**Rebuttal for PLOS Pathogens**

A. In response to the Reviewers’ comments from Review Commons

**Reviewer #1**

In this study, the authors investigated how HEV ORF2 interferes with host antiviral responses to sustain viral infection. They employed several models, including pattern recognition receptors (PRRs) KO cell lines, immunodeficient cells and stem cell-derived models, to prove that: 1) ORF2 is essential for viral replication and 2) ORF2 dampens the interferon and inflammatory signaling pathways. They confirmed the interaction between ORF2 and TBK1, a central mediator of innate immune responses and identified residues in ORF2 that affect its interaction with TBK1. Finally, through single cell RNAseq, they demonstrated that ORF2 is a viral antagonist that inhibits host ISG expression in both infected and bystander cells. Interestingly, the sets of genes that are upregulated in WT vs ORF2-deficient virus infected cells are not entirely identical, suggesting that ORF2 may also modulate host gene expression in addition to suppressing the immune response. This research confers new immune antagonism mechanisms mediated by HEV capsid for sustainable HEV replication in host cells and provides potential therapeutic targets for HEV treatments.

#### **Significance**

The study addresses a long-standing question in the field about the immune antagonism activities of HEV ORF2 and ORF3 which previous studies have conflicting results on. The strength of this study is the use of complementary approaches such as ORF2 trans complementation system and single cell sequencing, and more relevant models such as stem cell derived hepatocytes to rigorously dissect the role of newly synthesized ORF2 protein in immunocompetent cell context. The manuscript is well written and would appeal to researchers in the HEV and innate immunity fields. However, the significance of the study is dampened by changes in the IFN response not always correlate with the inconsistency of ORF2-mediated inhibitory effects in different models and the still poorly defined mechanism of ORF2 suppression of the IFN pathway. The study would make conceptual advance if the authors can address the discrepancies in their findings and perform additional characterization to determine the functional consequences of ORF2-mediated immune suppression and gene regulation.

My expertise is in innate immunity and host-virus interactions.

We thank the Reviewer for their positive evaluation of our study. Their very helpful and constructive comments have allowed us to improve the manuscript.

Major comments:

Comment 1a:

1a). The authors conclude from Figure 1 that the HEV ORF2 protein antagonizes both antiviral and inflammatory signaling pathways. The authors comprehensively investigated PRRs-mediated activation of type I interferon by viruses or poly(I:C) through overexpression of MDA5, RIG-I and TLR3. However, they only investigated the impact of ORF2 on host inflammatory response through evaluating the levels of TNFAIP3 RNA in the presence of MDA5 overexpression. It would be informative if the authors also check for NFkB activation/phosphorylation and expression of classical pro-inflammatory cytokines such as IL-1b and IL6.

As suggested by the Reviewer, we have also analyzed the impact of ORF2 on *TNFAIP3* and *IL6* expression in MDA5-overexpressing cells stimulated by poly(I:C) electroporation. Similar to our findings with TNF stimulation, we found that the presence of ORF2 reduces the strength of *TNFAIP3* and *IL6* expression. This data has been added to new Suppl. fig. 2 (mentioned in lines 166–169). Expression of *IL-1b* could not be analyzed because basal expression and expression upon stimulation was low in our cell line. As previously demonstrated, *TNFAIP3* is a direct response gene of NF-κB signaling, independent of IRF3 and IFN signaling (Wüst *et al.*, 2021, Cells). Therefore, we do not believe that analyzing NF-κB (p65) phosphorylation by Western blot would alter our conclusions.

Comment 1b:

Interestingly, changes in IFNB secretion after ORF2 overexpression appear more dramatic compared to changes in IFNB1 RNA levels (compare Figure 1A-C with Supplementary Figure 1A and C). Are the IFN-beta protein expression changes statistically significant in Supplementary Figure 1?

The changes in protein expression (absolute values) are likely to appear more dramatic due to the linear scale as opposed to the logarithmic scale used in Fig. 1A-C (relative values normalized to housekeeping gene expression). We have calculated the fold reduction in gene expression in cells expressing HEV-3 ORF2 and HEV-1 ORF2 relative to cells expressing GFP and added this information to both figures (line 933 and Supplementary Information, lines 285-286) and to the Results section (line 155-156), showing that the differences are similar. In addition, we have added information on the statistical differences in new Suppl. fig. 1C-E. As shown in this updated figure, the changes in IFNβ protein secretion are also significant when ORF2 is ectopically expressed (lines 158-160).

Comment 2a:

Changes in the IFN response do not always translate into changes in the viral RNA levels. In Figure 2B-D, the authors attributed the higher induction of IFNL1 and ISG15 on day 5 to the absence of ORF2 inhibition. However, the expression of these two genes drops to the same levels as the ones in WT viral RNA-electroporated cells on day 7, which is strange as ORF2-deficient viral RNA levels continue to be inhibited on day 7. This is different from the stem cell derived hepatocytes infected with the trans-complementation viruses in Figure 3G-H where there are significant differences in ISG15 levels between WT and ORF2-deficient virus infected cells on both days 5 and 7.

Indeed, we believe that by day 7, viral replication, regardless of which virus (WT or ΔORF2), induces a cell-intrinsic antiviral response that equilibrates to a level where both replication and the response can coexist. In Fig. 2B-D, ΔORF2 replication is dampened to a level where it induces an apparently similar response to WT replication. However, as shown in Suppl. fig. 4A-B, when the response is normalized to the number of replicating ΔORF2 genomes, the induced response is relatively higher due to the absence of the ORF2-mediated antagonism. We have added a statement in the revised manuscript for clarification (lines 219-221).

Regarding the apparent discrepancy observed in HLCs, we have also analyzed other genes besides *ISG15*, namely *IFNL1* and *IFIT1*, and found that these genes are induced to a similar extent by both WT and ΔORF2 infection of HLCs on day 7. We are now showing these data in the new Suppl. fig. 9D-E. As we show *ISG15* as a representative ISG throughout the manuscript, we would prefer to keep this panel in the main figures. However, we have explicitly added a statement in the revised manuscript stating that there are no apparent differences in the induction of *IFIT1* and *IFNL1* levels on day 7 between WT and ΔORF2 infection (lines 339-342 and Supplementary Information, lines 395-399).

Comment 2b:

To support their hypothesis, the authors need to further confirm the sudden upregulated antiviral activity on day 5 in electroporated HepG2/C3A cells by testing JAK/STAT phosphorylation and type I interferon secretion.

We thank the Reviewer for this suggestion and have analyzed IFN secretion as well as STAT1 phosphorylation on day 5 post-EPO accordingly. As shown in the new Suppl. fig. 5A (mentioned in lines 228-232 and Supplementary Information, lines 339-349), we measured the levels of secreted IFNα2A, IFNβ, IFNγ, and IFNλ1 in cell culture supernatants from WT- and ΔORF2-electroporated cells using a multiplex immunoassay (U-PLEX Interferon Combo (human), Meso Scale Discovery). Consistent with previously published work (Wu & Dao Thi et al., 2018, Gastroenterology), we did not detect any type I IFN secretion or type II IFNγ secretion. In contrast, we detected secreted type III IFN (IFNλ1) on all days post-EPO for both WT and ΔORF2. IFNλ1 levels were higher for ΔORF2 than for WT on all days post-EPO, which was significant on days 5 and 7 post-EPO.

In Suppl. fig. 5B (mentioned in lines 232-234 and Supplementary Information, lines 346-348), we further analyzed STAT1 phosphorylation by Western blot. Phosphorylated STAT1, but not basal STAT1 levels, were considerably higher on days 1, 5, and 7 post-EPO in the ΔORF2-electroporated samples than in WT.

Together, these findings further corroborate the upregulation of IFN and ISG induction on day 5 post-EPO that we initially observed in Fig. 2C and 2D. Furthermore, these data clearly indicate a stronger antiviral response also on day 7 post-EPO, which was previously only detectable upon normalization of the antiviral response over HEV RNA (Suppl. fig 4A-B). Respective methods have been added to lines 609-613.

Comment 3a:

The authors used different hepatocyte systems coupled with viral RNA electroporation or trans-complementation virus infection to investigate ORF2-mediated interference of the IFN pathway, which is highly complementary. However, while the electroporation of viral RNA into HepG2/C3A (Figure 2B-D) and infection of stem cell-derived hepatocytes with trans-complementation viruses (Figure 3F-H) result in similar upregulation of ISG expression on day 5, that wasn't observed in HepG2/C3A cells infected with trans-complementation viruses (Figure 3C-E) on day 5. The authors need to discuss the discrepancy among these different systems.

As the Reviewer points out correctly, due to the different routes of viral genome delivery (e.g., electroporation vs. authentic infection with full-length virus vs. synchronized and non-synchronized infection with trans-complemented ΔORF2 virus) and the different cellular backgrounds used (HepG2/C3A vs. HLCs), direct comparisons, especially with respect to temporal dynamics, are difficult to make. Therefore, we are careful not to make them in our manuscript.

We used electroporation to deliver WT, ΔORF2, or ΔORF3 genomes into cells, bypassing the cell entry steps. This allowed us to directly compare and determine the viral protein involved in enabling efficient viral replication. We have made this clearer in the Results section (lines 206-209).

Having developed a specific assay that allowed us to study the dynamics of HEV cell entry, we previously discovered that the process leading to successful delivery of the genome into the cytoplasm is rather slow for HEV compared to other viruses (Fu *et al.*, 2025, Nat Commun). In fact, the majority of infectious naked HEV particles do not uncoat until 12 h after entry (Fig. 4, Fu *et al.,* 2025, Nat Commun). Since electroporation bypasses this step, it is not possible to compare the kinetics of replication and the cell-intrinsic antiviral response following electroporated vs. infection-mediated delivery of the genome.

In addition, we believe that the requirements for the initiation of successful replication are likely facilitated in HepG2/C3A cells compared to HLCs. As already observed in our previous publication (Fig. 3 in Wu & Dao Thi *et al.*, 2018, Gastroenterology), the onset of replication occurs on day 3 post-infection in HLCs, which is perfectly consistent with the observations in the present study by Mehnert *et al.* (Fig. 3G, H). In HepG2/C3A cells, the onset of replication occurs earlier, already by day 2, which is in agreement with the study by Yin *et al.*, 2018, PLoS Pathogens (Fig. 1B). Therefore, in the present study, we strictly avoid comparing the kinetics of replication and cell-intrinsic antiviral response over time between the different systems used.

Why replication occurs earlier in HepG2/C3A is unclear but may be facilitated by a host factor that is absent in HLCs or by the presence of a host restriction factor (such as an ISG) that is present in HLCs. The identification of such factors should be addressed in future studies and we have added this aspect to the discussion section (lines 485-488). We have also added a more detailed explanation of the different culture systems used for clarification purposes (lines 479-485). However, we appreciate the Reviewers' acknowledgement that these systems are highly complementary, although they do not allow direct comparisons of viral replication kinetics.

Comment 3b:

Since the ORF2-deficient trans-complementation virus still brings in ORF2 proteins from the producer cells but cannot generate new ORF2 proteins, do ORF2 proteins from these two different sources have different functions in different hepatocyte systems? In addition, other than the data points that are shown to be not significantly different in Figure 3D-E, are any of the other data points significantly different?

This is a good point. In our recent publication, we have found that HEV enters cells through the lysosome and that cathepsin inhibitors prevent HEV infection (Fig. 5 and 6F, Klöhn *et al.*, 2024, Hepatology). We further confirmed that the capsid is likely degraded in the lysosome upon entry (Fig. 4 and 7, Fu *et al.*, 2025, Nat Commun). Therefore, it is unlikely that the ORF2 protein itself is being delivered to the cytosol of the infected cell from the incoming trans-complemented particles or from the WT particles. Consequently, we do not expect any differences in the function or activity of either ectopic ORF2 (expressed only in the producer cells) or ORF2 expressed by viral replication.

In Fig. 3C, the replication between WT and ΔORF2 is significantly different following the bottleneck at 56 h post-infection. Although ΔORF2 replicates at much lower levels than WT (Fig. 3C and Suppl. fig. 9A), this leads to a similar induction of *IFNL1* and *ISG15* (Fig. 3D, E). When the response is normalized to viral replication, as shown in Suppl. fig. 9B-C, it becomes clearer that the ΔORF2 virus induces a significantly higher response per replicating genome than the WT virus. We have made this clearer in the revised Result section of the manuscript (lines 330-332). Although, the scRNA-seq analysis clearly demonstrated that per infected cell, ΔORF2 induces a higher response than the WT (also see comment 5 to Reviewer #2 from PLoS Pathogens), we prefer to keep the non-normalized representation of the data in the main figures and the normalized data in the Supplementary figures. We made it clearer in the text that the normalized data can be found in the supplements (lines 328 and 332).

Comment 4a:

The single cell RNAseq data are very informative and revealed two interesting groups of genes. First, the ISGs that are further induced in the cells infected with ORF2-deficient HEV compared to cells infected with WT HEV (Figure 4N) are likely suppressed by ORF2. Second, the ISGs that are uniquely induced in the absence of ORF2 are different from the genes that are uniquely induced by WT HEV (Supplementary Table 2), suggesting that ORF2 may also modulate host gene expression.

We thank the Reviewer for acknowledging this analysis and for pointing out a very interesting aspect. Indeed, it appears that ORF2 not only modulates the cell-intrinsic antiviral response by antagonizing TBK1, as shown in this study, might also have a direct role in modulating host gene expression. A previous study reported that ORF2 harbors an NLS sequence and is translocated into the nucleus. The nuclear form of ORF2 was suggested to influence cytokine expression in infected cells (Suppl. fig. 6 of Hervouet *et al.*, 2022, PLoS Pathogens). While we have not yet confirmed these observations, many viral proteins are able to translocate into the nucleus and thereby exert different effects on host transcription by, for example, targeting transcriptional machinery or by altering chromatin states (reviewed in Liu *et al.*, 2020, Cell). As a result of this very constructive comment, we have already expanded on this aspect in our discussion section (lines 463-469) and now explicitly discuss the potential of ORF2 not only to modulate ISG expression by antagonizing TBK1 but also to affect host gene expression directly. We and others need to extend these observations and better characterize the impact of nuclear ORF2 on host gene expression.

Following this Reviewer’s suggestion, we have revisited our scRNA-seq data to more closely investigate the differences in the differential gene expression between infected cells of WT and ΔORF2 infections. We have generated a scatter plot showing a direct comparison of the differential expression of ISGs and other genes between WT and ΔORF2 infections. As we believe that the newly generated scatter plot is more informative than the previous Venn diagram, we have replaced it as new Fig. 4K (mentioned in lines 394-402 and 1015-1019). Among the most prominent and specifically upregulated genes in ΔORF2- vs. WT-infected cells are some of the ISGs from our ISG list (circled in black), highlighting again that the major differences are found in the antiviral response. We applied a threshold based on the distance from the diagonal to highlight genes with potentially higher expression in WT (Fig 4K, black) and ΔORF2 infection (Fig. 4K, magenta). We performed gene set enrichment analysis on these groups of genes but did not detect any enrichment (see our reply to comment 4c below).

Furthermore, due to the limited sequencing depth, we believe that more subtle differences in gene expression between WT and ΔORF2 infection are masked. Therefore, ORF2 may have additional functions and potentially modulates the expression of genes other than ISGs, either directly or indirectly. The groups of genes highlighted in black and magenta in our scatter plot (Fig. 4K) warrant further study, particularly in the context of the nuclear ORF2 isoform. However, this is beyond the scope of our study.

Comment 4b:

The authors can further characterize these two groups of ISGs by performing gene knockdown or knockout and investigating whether ORF2 directly interacts with these ISG products to determine the functional consequences of their upregulation.

This is a very good suggestion. However, we would prefer not to perform such an experiment at this stage as our collaborators have already performed an ISG screen based on the Schoggins library (Schoggins *et al.*, 2011, Nature) and will be publishing these results soon. In addition, since the amount of additional work (siRNA screen, co-IPs, etc.) would heavily delay the publication of our manuscript, we would prefer to not conduct this analysis at this stage. However, we fully agree that this is an interesting aspect that should be followed up on in the future. We have added this aspect to the Discussion section, as already detailed above (lines 463-469).

Comment 4c:

Related to that, are there other gene expression changes beyond ISG signatures which would suggest that ORF2 can regulate host gene expression? Figure 4A-C only shows comparisons for WT or ORF2-deficient vs. uninfected cells. The authors can perform GO and KEGG analyses to see if certain biological processes/pathways are enriched among the WT vs ORF2-deficient HEV induced genes. Further characterization of these genes (ISGs or not) would shed light on the novel roles of ORF2 in both immune antagonism and gene regulation and greatly increase the significance of the study

This is an excellent point, and we thank the Reviewer for bringing it to our attention. Accordingly, we have performed a gene set enrichment analysis of ΔORF2 vs. WT samples at the 56 h time point and added it to the revised Supplementary Information as new Suppl. fig. 10A (mentioned in lines 364-367 and Supplementary Information, lines 403-405). This analysis revealed that differences in the ISG response and metabolic changes are most significantly affected by ΔORF2 infection compared to WT infection.

As detailed in our response above (comment 4a), we revisited our scRNA-seq data to more closely examine the differences in differential gene expression between WT and ΔORF2 infections. While ISGs are among the most significantly and prominently upregulated genes in ΔORF2 compared to WT infection, other genes may also be directly or indirectly affected by ORF2. However, a detailed analysis of these genes and ORF2's role in their expression and/or regulation is beyond the scope of our study. Future studies should investigate the potential of ORF2 to regulate gene expression, especially in the context of the nuclear ORF2 isoform as discussed in the new version of our manuscript.

Comment 5:

In Supplementary Figure 3F-H, the authors used BX795 to inhibit TBK1 (a target of ORF2) and found decreases in IFNL1 and ISG15 expression whether cells are electroporated with WT, ORF2-deficient, or ORF3-deficient viral RNA. However, this does not correlate with the data in Figure 2E-G where TBK1 inhibition results in significant differences in viral RNA levels only in the absence of ORF2 or ORF3. These results would suggest that the effects of TBK1 inhibition on viral RNA levels is independent of changes in the IFN/ISG expression levels.

Indeed, the TBKi treatment leads to a similar reduction of *ISG15* expression in all samples but has a stronger effect on ΔORF2 replication compared to WT or ΔORF3, which we believe aligns perfectly with our discovery that ORF2, on the one hand, antagonizes the cell-intrinsic antiviral response (Fig. 1), and on the other hand, shields viral replication from the action of antiviral effectors (Fig. 2J). In contrast, WT and ΔORF3 replication are less vulnerable to the action of antiviral effectors and are therefore not significantly enhanced when TBK1 is exogenously blocked, even though the treatment leads to a decrease in ISG expression. Therefore, this observation introduces for the first time an additional protective function of ORF2 that we further confirmed in Fig. 2J.

The data shown in Suppl. fig. 4F-H served as a control to show the potency of TBK1 inhibition to exogenously reduce the virus-induced ISG response in cells electroporated with WT and mutant HEV genomes. Therefore, the same cell lysates were used for Suppl. fig. 4F-H and Fig. 2E-G, and we have added this information explicitly to the figure legend (lines 332-333 of Suppl. fig. 4 in the Supplementary Information). We have further added information on the fold changes between DMSO and TBKi-treated cells in Suppl. fig. 4F-H, which shows that on day 5 post-EPO, ΔORF2 induces a stronger response than WT or ΔORF3 in the absence of TBKi treatment, which can also be seen in Fig. 2C-D. It further highlights that the TBK1 inhibitor treatment reduces the antiviral response to a similar level for WT, ΔORF2 and ΔORF3. Overall, this substantiates our observation that inhibition of the antiviral response enhances ΔORF2 replication more significantly than WT or ΔORF3 replication, suggesting that ΔORF2 replication is more vulnerable to the action of ISGs. We have adapted the revised Result section to make these observations clearer (lines 252-253 and lines 277-278).

**Reviewer #2**

Authors described the protective mechanism mediated by ORF2 that protects viral replication from the antiviral responses. They have utilized the advanced single-cell RNA sequencing to decipher the dampened antiviral responses in the presence of ORF2 HEV. I believe the study is important for the HEV literature and believe that the manuscript can be considered for publication after authors (1) rewrites the results and discussions separately until the journal wants it to be together.

#### **Significance**

The paper uses advanced technique as single cell RNA seq to understand the mechanism of ORF2 assisting in the HEV replication.

The study is well designed.

This study will add up to understand some of the persistence infection seen in solid organ transplant patients. This study gives a mechanistic overview of HEV avoidance of antiviral response.

We thank the Reviewer for their positive evaluation of our study. We have addressed their helpful comments in the revised manuscript.

Major comments:

Comment 1:

Did the authors study only the P6 HEV genome? Have they done anything comparative with the other strain to understand if the proposed mechanism is not the strain specific?

We would like to thank the Reviewer for this interesting suggestion. Indeed, we have analyzed the behavior of an additional HEV-3 strain, the 83-2-27 strain (Shiota *et al.*, 2013, J Virol), which does not harbor any insertions in the HVR and has not been specifically adapted to cell culture. We have added this data as a new Suppl. fig. 6 (Supplementary Information, lines 352-359; also in response to Reviewer 3, Review Commons), mentioned in lines 237-243.

Upon electroporation of 83-2-27 WT and ΔORF2 into HepG2/C3A cells, we observed significantly lower HEV RNA levels for ΔORF2 on day 7 post-EPO compared to WT (Suppl. fig. 6A). ΔORF2 replication induced stronger IG15 expression compared to WT replication on all days post-EPO; however, this difference was only significant on day 3 (Suppl. fig. 6B). The differences in the kinetics of the ΔORF2 phenotype may result from differences in the replication kinetics and the overall lower replication of the 83-2-27 compared to the Kernow-C1/p6 strain.

Nonetheless, we were able to validate the observations made for the cell culture-adapted HEV-3 Kernow-C1/p6 strain with a different, non-cell culture-adapted HEV-3 strain.

Comment 2:

Can the authors explain why we do not see any band in the Fig. 1F B-actin?

We do not expect to detect β-actin in our IP samples as no specific interaction between ORF2 and β-actin has been reported so far. Therefore, the lack of a β-actin band in our sample after the IP highlights the reliability and accuracy of the assay, and moreover, it underlines the specificity of the interaction between ORF2 and TBK1.

Minor comments:

Line 69, 71 - I have never seen in any paper including reference in this way!

We removed the respective citation style, as suggested, to make the manuscript more appealing to the reader.

Line 72 and 73 - missing reference!

We added the missing reference.

Line 92, 93 - missing reference!

We added the missing reference.

Line 95 to 99 - missing references!

We added the missing reference.

**Reviewer #3**

**Significance**

In the manuscript entitled " The hepatitis E virus capsid protein ORF2 counteracts cell-intrinsic antiviral responses to enable persistence in hepatocytes ", Ann-Kathrin Mehnert interrogated that HEV pORF2 can inhibit host antiviral response. They found interaction of HEV ORF2 and TBK1. The finding is interesting and echoed with some previous studies that ORF2 can inhibit innate immunity.

The study could benefit from the consideration of some major and specific points, as indicated below.

We thank the Reviewer for their appreciation of our study. We have addressed all comments which we considered helpful and very constructive.

Major comments:

Comment 1:

The researchers used p6, a cell-adapted clone, which was isolated form a chronic HEV patient. As previous studies suggested, p6 may behave differently than wild-type strains. Did the authors tried other HEV strains, as they used ips-induced model that was reported supportive to wild-type HEV?

As already detailed in the response to Comment 1 from Reviewer 2 (Review Commons), we generated a ΔORF2 mutant of the HEV-3 83-2-27 strain, which is devoid of insertions in the HVR or any other mutations and has not been adapted to cell culture. We analyzed HEV replication and *ISG15* expression upon electroporation of 83-2-27 WT and ΔORF2 in HepG2/C3A cells and, similarly to the HEV-3 Kernow-C1/p6 strain, also observed a decreased replication and stronger *ISG15* induction for ΔORF2 compared to WT. This corroborated that the previously observed ΔORF2 phenotype is not specific to the Kernow-C1/p6 strain (mentioned in lines 237-243).

Fig. 1 of our manuscript demonstrated that ORF2 proteins from both HEV-3 and HEV-1 are, in principle, able to interfere with antiviral and inflammatory signaling. However, we have shown previously that infections with primary isolates from different genotypes result in varying viral replication and antiviral response kinetics (Wu & Dao Thi, 2018, Gastroenterology). Even though cDNA clones of HEV-1 (e.g. Sar55 strain) exist, these differences in replication and antiviral response kinetics might impede conclusive interpretations when comparing WT and ΔORF2 mutants between HEV-1 and HEV-3 strains. In addition, we have unfortunately not yet optimized the delivery of HEV genomes to HLCs via transfection. They are hyper-responsive and often de-differentiate upon transfection of viral genomes, impeding conclusive results. In our manuscript, we have infected HLCs with trans-complemented HEV-3 particles (Fig. 3G-H), but these are impossible to produce at this stage for HEV-1 as this genotype does not replicate in hepatoma cells.

For all of the abovementioned reasons, we hope the Reviewer understands that we decided to solely focus our attention in our full-length infectious systems on HEV-3, comparing the Kernow-C1/p6 and 83-2-27 strains in HepG2/C3A cells, which is the cell line used for the majority of our study.

Comment 2:

Figure 1F, ORF2 can interact with TBK1 as showed. But the prediction from Alphafold is weak. Also, could the author more evidence than the co-IP?

AlphaFold has recently emerged as a very powerful and important tool for predicting protein-protein interactions. However, our collaborator Dr. Thibault Tubiana, who performed the predictions for us, has found that AlphaFold does not perform well when predicting interactions with large proteins. Therefore, we only modeled the potential interactions between TBK1 and individual segments of the ORF2 protein (N-terminus, S-, M-, or P-domain). We could not identify a reliable interaction, although the low PAE scores (as shown in Suppl. fig. 3E) do not fully rule out that the full-length ORF2 protein still interacts with the TBK1 dimer. As we discuss in our manuscript (lines 508-513), it is also possible that the interaction occurs with the unstructured ORF2 N-terminus, and thus modeling the interaction with AlphaFold is likely to be challenging.

In order to confirm the ORF2-TBK1 interaction aside from the IP shown in Fig. 1F, we also performed co-localization studies based on immunofluorescence staining and confocal microscopy. As shown in Revision figure 1, we observed a strong co-localization between ectopically expressed TBK1 and large ORF2-positive structures, which further corroborates their interaction. It should be noted that for this analysis, we had to detect V5-tagged TBK1 via V5 staining due to the lack of appropriate antibodies capable of detecting endogenous TBK1.

However, we believe that the stringent conditions of the co-IP presented in our manuscript more convincingly support a direct interaction between ORF2 and TBK1 than co-localization studies by immunofluorescence staining. The interaction between ORF2 and TBK1 has already been published previously (Lin *et al.*, 2019, Viruses), and our data presented here is in agreement with this study. Furthermore, we are still in the process of studying and characterizing the large ORF2-derived structures observed by immunofluorescence, as seen in Revision figure 1. As this is part of current and future work in our research group, we prefer not to show these images in our manuscript. However, we believe they further consolidate the interaction between ORF2 and TBK1.

**Revision figure 1: ORF2 co-localizes with TBK1.**

S10-3 cells were electroporated with *in vitro* transcribed HEV WT RNA. After 4 days, the cells were transfected with a plasmid encoding TBK1-V5. On day 2 post-transfection, the cells were fixed with 2% PFA. The cells were treated with cold methanol for 20 min, blocked and permeabilized with 10% goat serum/1% BSA/0.1% Triton X-100 in PBS for 1 h, and incubated with primary antibodies against ORF2 (1:400, Merck, MAB8002) and V5 tag (1:1000, Cell Signaling Technology, #13202) at 4 °C overnight. The cells were incubated with fluorophore-conjugated secondary antibodies (1:1000) and Hoechst 33342 (1:1000) for 1 h at room temperature. Images were taken with a Zeiss LSM900 Airyscan 2 microscope. Z stacks have a 0.3 μm interval. Maximum projection images are shown. Scale bar, 10 µm.

Comment 3:

Figure 2C and 2D, at 5 dpi, one can observed a stronger antiviral response, but at 7 dpi, no obvious difference was observed. Could the authors comment on this?

Indeed, and as clarified in our response to Reviewer #1 (Comment 2a), we believe that around day 5 post-EPO, ΔORF2 induces a strong antiviral response that subsequently leads to a decline in viral replication. By day 7 post-EPO, similar to WT, ΔORF2 replication and the antiviral response equilibrate to a level where both can coexist. However, ΔORF2 replication is more dampened compared to WT replication due to the lack of the TBK1 antagonism and protection from antiviral effectors. We have clarified this in the updated Results section (lines 219-221, 234-236, and 292-294).

Comment 4:

Figure 2H and 2I, detailed description of how the authors measured the positive cells should be provided. Did the authors selected whole plate of cells for counting? As showed in Figure 2H, the signals of IF were stronger at 5 and 7 dpi when compared at 3 dpi, but why the proportion of positive cells was reduced in Figure 2I?

We apologize for the confusion. The images shown in Fig. 2H are not based on IF staining but on RNA-FISH, a method that uses fluorescent probes to detect individual HEV genomes (Fu *et al.*, 2025, Nat Commun). Due to the absence of the viral proteins ORF2 or ORF3 in the ΔORF2 and ΔORF3 mutants, respectively, we decided to quantify infected cells based on the detection of viral genomes, allowing the direct comparison of the mutants with WT. Therefore, an increase in signal per cell indicates an increase in replication per cell over time, which is why the signals are stronger on days 5 and 7 compared to day 3 (Fig. 2H). The observation that the number of positive cells slightly decreases over time for WT and ΔORF3 indicates that the antiviral response is likely able to limit not only the replication per cell but also affects the percentage of infected cells. For the ΔORF2 virus, both the replication per cell and the number of replicating cells decrease significantly over time, highlighting the critical role of ORF2 in providing HEV with the ability to persistently replicate. We have made the use of RNA-FISH for this analysis more explicit in the Results section of the revised manuscript (lines 255-263).

In addition, we have added the information that we used five randomly selected fields of view per condition and biological replicate for the quantification to the updated figure legend of Fig. 2 (lines 965-966) and in the Supplementary Methods (“RNA fluorescence in situ hybridization and quantification”, lines 57-58). We have also specified in the figure legend that the positive cells were quantified with ilastik and CellProfiler (lines 964-965).

Comment 5:

The study emphasized the function of ORF2 on HEV "persistence". However, this cannot be fully supported by cell models. In future, study on chronic HEV infection animal models may be conducted.

Recent discoveries of inborn errors of immunity have taught us that the cell-intrinsic innate immune response is critical for limiting pathogen infections. In the present manuscript, we have investigated how HEV achieves persistent viral replication in *in vitro* cell culture models. We believe that we have provided strong evidence that in the absence of the capsid protein ORF2, HEV replication cannot be sustained due to the efficient cell-intrinsic antiviral response.

Since we are fully aware that cell culture models based on monocultures of hepatocytes are not sufficient to identify the determinants that lead to viral persistence and chronicity *in vivo,* we refrain from making any such claims. Instead, we and others strongly believe that infected hepatocytes affect professional innate immune cells, which in turn are critical for efficient B- and T-cell responses *in vivo*. Therefore, a detailed understanding of the viral interference with the cell-intrinsic antiviral response constitutes an important basis for more complex studies. We thus propose to consolidate our findings in more complex models, e.g. based on co-culture of infected hepatocytes and innate immune cells, for example pDCs. We have now explicitly added the limitations of our study together with the notion that in the future, our findings on ORF2 should also be complemented and consolidated in immunocompetent animal models (lines 531-537), according to the Reviewer’s suggestion.

Comment 6:

The authors study ORF2 in whole. It will be of benefit to the readers that the authors could specified the function of secreted ORF2 and ORF2 capsid in the current study.

This is a very interesting point. Indeed, we have previously tested the ability of the different isoforms of ORF2, namely intracellular ORF2i and glycosylated ORFg, to enable viral replication in cell culture. For this purpose, we mutated the respective start codon of each isoform in the full-length genome according to the strategy described by Yin *et al.*, 2018, PNAS (Revision figure 2A). Removal of the glycosylated form allowed the ORF2i mutant to replicate in the presence of a dampened antiviral response similar to WT. This suggested that the cytosolic isoform ORF2i is sufficient to inhibit TBK1 and shield viral replication from antiviral effectors.

However, when we attempted to remove the intracellular form to only retain ORF2g, we observed an intermediate phenotype. The ORF2g mutant replicated to lower levels compared to ORF2i and the WT, but not as low as ΔORF2 (Revision figure 2B). Similarly, it induced a stronger response than ORF2i and WT, but not as strong as ΔORF2 (Revision figure 2C-D). Ongoing work in our lab has shown that some of the ORF2g isoform is retained in the cytosol despite expression of the signal peptide. Since we are still in the process of consolidating this, and since it remains a matter of debate in the field how maturation and localization of the different ORF2 isoforms is regulated (Yin *et al.*, 2018, PNAS; Hervouet *et al.*, 2022, PLoS Pathogens), we prefer not to show these data in the manuscript. However, we acknowledge that this should be conclusively studied in the future, and we have included this aspect in the Discussion section of the revised manuscript (lines 495-498).

**Revision figure 2: The ORF2i isoform rescues the ΔORF2 phenotype.**

(A) HepG2/C3A cells were electroporated with IVT RNA of HEV WT (black), ΔORF2 (magenta), and full-length HEV mutants expressing only ORF2i (orange) or ORF2g (green). On day 7 post-EPO, cell lysates and supernatants were harvested, treated with PNGase F to remove *N*-glycosylations, and then analyzed by Western blot for protein expression of ORF2 and β-actin. Representative blot of n = 2 independent biological experiments. (B) HepG2/C3A cells electroporated as described in (A) were analyzed for HEV RNA by RT-qPCR. (C) *IFNL1* and (D) *IFIT1* expression were analyzed relative to *RPS11* and normalized to HEV RNA using the 2^-ΔΔCt^ method. Data show mean ± SEM of n = 2 independent biological experiments. A.U., arbitrary units; n/d, not detectable; norm., normalized.

Minor comments:

Minor comment 1:

Figure 3A, this is an elegant design. More data may provide for the validation of the formation of the virions.

In order to demonstrate the formation of infectious particles, we harvested ORF2-trans-complemented virions either intracellularly (naked) from the lysate or extracellularly (quasi-enveloped) from the supernatant of ΔORF2 RNA-electroporated S110-3/ORF2 cells. We then separated naked and quasi-enveloped HEV particles by density gradient ultracentrifugation. In each fraction of the gradient, we detected ORF2 capsid by Western blot analysis and HEV genomes by RT-qPCR at the expected densities. We have added these data as a new Suppl. fig. 8, mentioned in lines 302-308 and in the respective Supplementary Information (lines 155-164 and 374-381). We show that the presence of ORF2 protein and the peak of HEV RNA in fractions 9-10 (densities 1.2065 to 1.2576 g/mL) from the gradient separating intracellularly harvested particles confirmed the presence of ΔORF2-nHEV (Suppl. fig. 8A; buoyant density of 1.25 g/mL). ORF2 protein and HEV RNA in fractions 3-5 (densities 1.0454 to 1.1026 g/mL) from the gradient separating extracellularly harvested particles verified the production of ΔORF2-eHEV (Suppl. fig. 8B; buoyant density of 1.10 g/mL).

Therefore, we were able to validate the formation of infectious ΔORF2 virions and can conclude that ORF2 trans-complementation allows the completion of the full HEV life cycle.

Minor comment 2:

Figure 1, data should be provided for the successful expression of HEV-1 or HEV-3 ORF2, and ORF3.

We have analyzed the A549-derived cell lines for successful expression of HEV-1 and HEV-3 ORF2 as well as HEV-3 ORF3. We have added the respective Western blot analysis as panel A to the Suppl. fig. 1 (mentioned in lines 149-150 and Supplementary Information, lines 280-283).

Minor comment 3:

line 219, the current evidence that supported this statement is weak, especially for ORF2.

We have adjusted and toned down our interpretation in the manuscript, acknowledging that we can only make an assumption about the effect of secondary virus spread in our system. (lines 223-225).

Minor comment 4:

Suppl Figure 3F-3H, statistical analysis is needed

We have added the statistical analysis to Suppl. fig. 4F-H (previously Suppl. fig. 3F-H), also as described in our response to Comment 5 of Reviewer #1.

Minor comment 5:

Suppl Figure 3F-3H, it seems that when no treatment was admistrated, the level of ISG15 in ΔORF2 group was higher than those of the WT and ΔORF3 group. Could the authors comment?

Indeed, in absence of the TBKi treatment, electroporation with the ΔORF2 genome resulted in a stronger induction of *ISG15* on day 5 post-EPO compared to WT and ΔORF3. The data in Suppl. fig. 4F-H corresponds to the data shown in Fig. 2B-G, where this phenotype is already visible. We discuss in our manuscript that the lack of the TBK1 antagonism mediated by ORF2 probably causes the stronger induction of the antiviral response in the ΔORF2 mutant. We have added a statement in lines 234-236 to make this observation clearer to the reader.

Minor comment 6:

Figure 3D and 3E, the starting time of the detection is not aligned.

We apologize for the confusion. While *ISG15* expression is detectable at basal levels and already slightly increased 24 h post-internalization, *IFNL1* expression is not detectable at basal levels. We only detected *IFNL1* mRNA at 32 h post-internalization. For clarification, we added “n/d” (not detectable) to the *IFNL1* graph prior to the 32-hour time point to better align the graphs.

Minor comment 7:

Figure 3F, scale bar is missing.

We have added scale bars to all images in Fig. 2 and Fig. 3.

Minor comment 8:

In M&M, statistical method should be provided with more details and cover all the experiments used.

As different statistical tests had to be used for different experiments in our study, depending on the type of comparison to be made, we specified the statistical analysis used for each panel in the figure legends. We prefer to keep it this way as we believe that otherwise it will be difficult to assign the correct statistical analysis. We added a note to the Methods section clarifying that information on statistical tests and p-values is given in the respective figure legends (lines 688-689).

B. In response to the Reviewers’ comments from PLOS Pathogens

**Reviewer #1**

In this study, the authors have provided a comprehensive introduction of previous research on the role of HEV ORF2 and ORF3 in cell-intrinsic antiviral responses. They emphasized the major limitations of these previous studies in particular the use of overexpression approach of viral proteins, and the use of cell line based models. Therefore, the authors aimed to better clarify ORF2 and ORF3 proteins in antiviral response by: 1) use of viral mutants; 2) different immunocompetent hepatocellular systems. However, there are major issues in their claims and just to list a few below.

Major comments:

Comment 1:

Figure 1, the results are also based on ectopic overexpression of ORF2 and ORF3. By the way, overexpression per se is not a problem, but the authors challenged the previous studies of using this approach while they are still using this method.

In our manuscript, we did not challenge the approach of studying viral proteins by ectopic expression. In contrast, we aimed to validate these results using the same approach, as contradictory results had been previously published, as stated in lines 125-127 and 140–144. We did so within Fig. 1. Based on this ectopic expression study, we then extended our observations and confirmed the role of ORF2 in HEV replication, as the relevance of the antagonisms published by previous studies remained unclear in the context of full-length viral replication or infection.

Comment 2:

Figure 1, A549 cell line was used (a human lung carcinoma cell line);

Figure 2, HepG2/C3A cells used (derivative of Hep G2 cell line originally isolated from the liver of a patient with hepatocellular carcinoma);

Figure 3, S10-3 cells (a subclone of the human hepatoma Huh7 cell line) and HepG2/C3A cells, although human pluripotent stem cell-derived hepatocyte-like cells (HLCs) were also used;

Figure 4, HepG2/C3A cells used;

Overall, these models are not fundamentally different or superior to the models used in previous studies, and it is unclear how their models qualified as “immunocompetent hepatocellular systems”. Furthermore, in the title “The hepatitis E virus capsid protein ORF2 counteracts cell-intrinsic antiviral responses to enable persistence in hepatocytes”, the word “hepatocytes” is somewhat deceiving, suggesting the main findings are based on primary hepatocytes or at least stem cell-derived HLCs. In fact, most of the results were obtained from cell lines.

Throughout our manuscript, no claims were made regarding the superiority of the cellular systems used as compared to previous studies. In agreement with previous studies (Joshi *et al.*, 2025, Life Sci Alliance; Yin *et al.*, 2018, PLoS Pathogens), we identified HepG2/C3A cells as a suitable model for our study due to their permissiveness to HEV infection and the resulting induction of a cell-intrinsic antiviral response. We used a physiologically more relevant hepatocyte system (hESC-derived HLCs) only to validate our findings made with HepG2/C3A cells. In order to meet this Reviewer's concerns, we have carefully removed the word “immunocompetent”, for example in lines 34-35 and 46, as well as throughout our Results and Discussion sections.

We also understand the Reviewer’s concern about the title of the manuscript and have changed it to: "The hepatitis E virus capsid protein ORF2 counteracts cell-intrinsic antiviral responses to enable persistent replication in cell culture." Furthermore, in response to Review Commons Reviewer #3, Comment 5, we have added a section to our Discussion, acknowledging the limitations of our study, stating that persistent replication in cell culture is, of course, not equivalent to persistence *in vivo* (lines 530-536).

Comment 3:

Overall, most of the results are descriptive, and even segmented. It is unclear how their findings can further advance the current knowledge in the field.

We acknowledge the Reviewer's criticism. Nevertheless, we believe that our findings make a valuable contribution to advancing current knowledge in the field, a view that is supported by the positive and favorable feedback from the other four Reviewers. We believe that we have highlighted the advances sufficiently in our manuscript, for example in lines 192-195, 271-273, 291-293, 342-344, 411-414, 417-423, and 547-555.

Here, we would like to stress again the most important aspects: First, we have, for the first time, performed a comprehensive and side-by-side comparison of the potentials of ORF2 and ORF3 to interfere with IRF3-, NF-κB-, and IFN-mediated signaling. Furthermore, we have performed this comparison in a well-characterized cell culture system that allowed us to dissect signaling downstream of all relevant PRRs (MDA5, RIG-I, TLR3) separately. In contrast, previous publications have usually focused on a single viral protein. Second, we could show for the first time that the TBK1 antagonism mediated by ORF2 is relevant for the replication of a full-length HEV-3 strain, as a stronger antiviral response was induced in the absence of ORF2, resulting in decreased viral replication. Third, we identified an additional function of ORF2, shielding HEV RNA from the effects of the antiviral ISGs. Altogether, we showed that these ORF2-mediated immune evasion strategies are important for continued HEV replication following a bottleneck early in infection. And lastly, we performed, for the first time, scRNA-seq analysis of HEV-infected cells. We could highlight that the major changes in gene expression affect the ISG response and that both HEV-infected cells and bystanders appear to contribute equally to the ISG response.

Overall, we are convinced that our study significantly contributes to advancing the current understanding of the mechanisms that allow HEV replication to persist in cell culture despite the induction of an antiviral response.

**Reviewer #2**

Mehnert AK et al. are describing in their manuscript the potential role of the ORF2 capsid protein in counteracting the innate and inflammatory responses during HEV infection. The authors employed different cell culture models, i.e. A549, HepG2/C3A, Huh-7.5 and hepatocyte-like cells, together with full-length HEV genomes and infectious viruses. They complemented there investigations by a single cell RNA sequencing analysis of HepG2/C3A cells infected by wt or delORF2 HEV.

The study is taking advantage of original tools (KO cells, mutated full-length genomes) and advanced cell models or techniques (HLC, scRNA-seq) to tackle a very relevant question which has been addressed in the past but with inconsistency in the conclusions.

We would like to thank the Reviewer for their appreciation of our study and for their thoughtful suggestions to improve the structure and readability of our paper.

Major comments:

Comment 1a:

While the study is very interesting and revised concepts in light of advanced techniques, the current manuscript has somehow difficulty to convey a clear message. The manuscript may greatly gain in readability by splitting some of the figures as well as the results section into distinct paragraphs. In particular, the co-IP experiment showing TBK1 and ORF2 interaction is not well-integrated in Fig 1. The authors may want to reorganize it together with results of Fig 2 in which they show result with TBK1 inhibitor or simply omit this result. Figure 2 may also be divided.

We thank the Reviewer for this suggestion.

We believe it is important to demonstrate the reproducibility of previously published results. Therefore, we prefer to keep and display the TBK1/ORF2-IP. For the following reasons, however, we strongly believe that the TBK1/ORF2-IP should be included in Fig. 1, and we hope the Reviewer agrees:

Fig. 1 focuses entirely on mechanistic investigations of the ORF2 protein's antagonistic functions outside the replication context. This figure mechanistically shows how ORF2 interferes with antiviral signaling. Therefore, we believe that the co-IP belongs in this figure.

Fig. 2 focuses entirely on the role of ORF2 in viral replication. We used a commercially available TBK1 inhibitor to blunt the sensing pathway independently of the antagonism mediated by the interaction between ORF2 and TBK1. We have made this clearer in lines 244-247. Our goal was to suppress the sensing pathway and, consequently, the IFN expression and ISG responses induced by viral replication. This demonstrates that the ΔORF2 virus is more sensitive to the response and that replication can be restored through treatment.

Comment 1b:

In addition, the text may be streamlined to gain in interest. The authors are extensively justifying their experimental conditions which can be limited. As an example, lines 237-239, the authors explained why BX795 is employed only for 48h, but in my view this can be omitted in the main text as it overloads the results section and dilute the main information.

We agree with the Reviewer and have removed the explanation of the incubation time with the inhibitor BX795 (lines 245-246). We shortened other explanations as well, such as those in lines 363-366 and 277-278, to improve the readability of the Results section.

Minor comments:

Minor comment 1:

In Fig1, gene expression is related to GAPDH expression why in Fig 2 and 3 the authors used RPS11 for the normalization. Is there a rationale for this?

The results of Fig. 1 were produced in the research group of our collaborator, Dr. Marco Binder, where *GAPDH* is used as the standard housekeeping gene. In the Dao Thi laboratory, however, we prefer *RPS11* as our housekeeping gene. Therefore, experiments performed in A549-derived cell lines were analyzed using *GAPDH* (Fig. 1), and all other experiments were analyzed using *RPS11* (Figs. 2 and 3). There is no other underlying reason for this distinction.

Minor comment 2:

Title of Fig2 mentions “in immunocompetent cells“. I would remove this notion since panel J has been performed in Huh-7.5 cells.

As suggested, we have removed the phrase “in immunocompetent cells” from the figure legend of Fig. 2 (line 945) and from the title of the corresponding Results section (lines 198 and 199).

Minor comment 3:

L205-206: “Comparable infection with trans-complemented…“, in the context, the sentence is difficult to understand (trans-complementation system not introduced earlier) and provide complexity. This should be revised.

We would like to thank the Reviewer for this attentive observation. We have therefore removed the mention of “trans-complemented” in this context (lines 205-208), as trans-complementation is only introduced later on in the manuscript.

Minor comment 4:

L224-233: the authors are explaining the controls they used in the experiments. It would be better to integrate these informations within the description of the results rather than making this point at the end of the paragraph.

We understand the Reviewer’s concern. However, we are not entirely sure if the Reviewer refers to the information on the GNN control in these lines. We have mentioned this control actually already before in the paragraph (line 212-213) where we believe its description to be well integrated.

In the lines the Reviewer was referring to, we are rather interpreting our results. For example, we are using the lack of antiviral response induction in the GNN mutant to further understand the effect that we observed with the ΔORF2 mutant. Hence, the ΔORF2 phenotype needs to be described first before comparisons with the GNN mutant can be made.

Following this helpful comment of the Reviewer, however, we have decided that this paragraph is better placed within the Discussion section (lines 429-432) and have therefore removed the interpretation of the GNN mutant from the Results section.

Minor comment 5:

Fig 3E, ISG15 is not differentially expressed during infection of HepG2/C3A cells with delORF2 and wt HEV while in scRNA-seq analysis it seems significantly different.

This is indeed an interesting observation and we would like to thank the Reviewer for pointing it out. The differential gene expression values and/or fold changes between RT-qPCR of bulk populations vs. scRNA-seq analysis are not directly comparable. As shown by the scRNA-seq analysis, despite infecting with the same genome copy numbers per cell, we reached a higher infection rate with the WT (28.1%) than with the ΔORF2 (17.2%) virus at 56 h post-infection (Suppl. fig. 10D). In bulk analysis, these differences in the percentages of infected cells can result in seemingly equal ISG15 induction, even though the ISG15 expression per cell is in fact higher for ΔORF2 than for WT. This clearly highlights the advantages of our scRNA-seq analysis which enabled us to show that the ISG expression per cell was globally increased in infected cells as well as bystanders (due to the higher induction of IFN) upon ΔORF2 infection. We have made this observation clearer in lines 440-442.
